# Transcriptional and post-transcriptional control of autophagy and adipogenesis by YBX1

**DOI:** 10.1038/s41419-023-05564-y

**Published:** 2023-01-16

**Authors:** Ruifan Wu, Shengchun Feng, Fan Li, Gang Shu, Lina Wang, Ping Gao, Xiaotong Zhu, Canjun Zhu, Songbo Wang, Qingyan Jiang

**Affiliations:** Guangdong Laboratory of Lingnan Modern Agriculture, Guangdong Province Key Laboratory of Animal Nutritional Regulation and National Engineering Research Center for Breeding Swine Industry, College of Animal Science, South China Agricultural University, Guangzhou, 510642 China

**Keywords:** Macroautophagy, RNA decay, Differentiation, Transcription

## Abstract

Obesity is strongly associated with metabolic diseases, which have become a global health problem. Exploring the underlying mechanism of adipogenesis is crucial for the treatment of excess white fat. Oncogene YBX1 is a multifunctional DNA- and RNA-binding protein that regulates brown adipogenesis. However, the role of YBX1 in white adipogenesis and adipose tissue expansion remains unknown. Here, we showed that YBX1 deficiency inhibited murine and porcine adipocyte differentiation. YBX1 positively regulated adipogenesis through promoting ULK1- and ULK2-mediated autophagy. Mechanistically, we identified YBX1 serves as a 5-methylcytosine (m^5^C)-binding protein directly targeting m^5^C-containing *Ulk1* mRNA by using RNA immunoprecipitation. RNA decay assay further proved that YBX1 upregulated ULK1 expression though stabilizing its mRNA. Meanwhile, YBX1 promoted *Ulk2* transcription and expression as a transcription factor, thereby enhancing autophagy and adipogenesis. Importantly, YBX1 overexpression in white fat enhanced ULK1/ULK2-mediated autophagy and promoted adipose tissue expansion in mice. Collectively, these findings unveil the post-transcriptional and transcriptional mechanism and functional importance of YBX1 in autophagy and adipogenesis regulation, providing an attractive molecular target for therapies of obesity and metabolic diseases.

## Introduction

Obesity has been rapidly prevailed over the past several decades worldwide [1]. Epidemiologic studies have revealed a strong association between obesity and an extensive range of diseases such as type 2 diabetes mellitus, non-alcoholic fatty liver, cardiovascular disease, musculoskeletal diseases, mental disorders and cancer [2], which constitutes a public health issue of major proportion. At the cellular level, obesity is originally resulted from an increase in the fat cell number (hyperplasia) and/or the size (hypertrophy) of individual adipocytes. Thus, improving our understanding of the molecular mechanism of adipogenesis and identifying its novel regulators may lead to effective therapies for obesity and its associated metabolic disorders.

Macroautophagy (hereafter referred to as autophagy), a fundamental and evolutionarily conserved homeostatic process for cellular quality control through degradation and recycling of cellular components, has gained much attention as a therapeutic target of obesity [3, 4]. Previous studies showed that autophagy played a significant role in adipose tissue expansion and adipogenesis [5–8]. Autophagy was upregulated in adipocytes from obese patients or diabetic mice [9, 10]. Adipose-specific deletion of autophagy-related 7 (*Atg7*) prevented high-fat diet (HFD)-induced obesity and improved insulin sensitivity in mice [6]. ATG5 deficiency dramatically inhibited adipogenesis in vitro and in vivo [7]. The core autophagy regulators Unc-51–like kinases 1 and 2 (ULK1 and ULK2) belong to the ULK/Atg1 family of serine/threonine kinases, which are conserved from yeast to mammals [11]. ULK1 and ULK2 play a central role in autophagosome formation and autophagy induction in various cell types [8]. Autophagy deficiency caused by ULK1 or ULK2 dysregulation has been implicated in numerous diseases, including cancer [12, 13], diabetic disease [14], chronic kidney disease [15], and cardiomyopathy [16]. A previous study found that ULK2 was required for adipocyte differentiation, while ULK1 is dispensable for adipogenesis [8]. However, recent studies reported that estradiol signaling, sirtuin 1 (SIRT1) pathway and AMP-activated protein kinase (AMPK) inhibition suppressed ULK1, thereby inhibiting autophagy and adipogenesis [17–19], suggesting a regulatory role of ULK1 in adipogenesis. Thus, the role and upstream regulator of ULK1 and ULK2-mediated autophagy in adipogenesis regulation are still unclear.

Y-box binding protein 1 (YBX1) is a multifunctional DNA- and RNA-binding protein belonging to a large family of proteins with an evolutionarily conserved cold shock domain (CSD) [20]. Functionally, YBX1 modulates diverse DNA/RNA-dependent process such as transcription, DNA reparation, mRNA stability, splicing, and translation [20–22]. At the cellular level, YBX1 participates in various important cellular processes, including cell proliferation, differentiation, autophagy, stress response and carcinogenesis [20, 23, 24]. Notably, recent studies identified YBX1 as a cytoplasmic mRNA 5-methylcytosine (m^5^C) reader protein [25, 26]. m^5^C is a prevalent modification in RNAs and has been reported to play a critical role in RNA metabolic regulation [27]. Emerging evidence showed that YBX1-mediated m^5^C was involved in cancer pathogenesis and development [28]. A recent study found that YBX1 knockdown inhibited white preadipocytes commit to beige lineage and impaired thermogenic potential at the transcriptional level [29]. Our previous study also showed that YBX1 regulated thermogenesis and brown adipogenesis through mitophagy [30]. However, the role of YBX1 in white adipogenesis and adipose tissue expansion is still a mystery. Furthermore, the relationship between YBX1 and autophagy in adipocytes remains unknown.

In this study, we investigated the role and function of YBX1 in adipogenesis and autophagy by using two ideal cell model, mouse 3T3-L1 cell line and porcine primary preadipocytes. 3T3-L1 is a well-established preadipose cell line as the standard model system to identify regulators of adipogenesis and fat cell function [31]. Porcine preadipocytes are an excellent model for studying human developmental processes and congenital disease, because of their high similarity to human cells in physiology, immunology and genome [32]. We demonstrated that YBX1 facilitated adipogenesis through enhancing ULK1- and ULK2-mediated autophagy. Further study showed that YBX1 modulated autophagy by post-transcriptional control of *Ulk1* in an m^5^C-dependent manner and transcriptional regulation of *Ulk2*. In addition, YBX1 overexpression in white adipose tissue (WAT) enhanced autophagy and promoted adipose tissue expansion in mice. Our study revealed that YBX1 played a significant role in the regulation of autophagy and adipogenesis, providing a promising target to fight against obesity and related metabolic diseases.

## Materials and methods

### Cell culture and adipocyte differentiation

The mouse 3T3-L1 preadipocytes used in this study were purchased from ATCC. The porcine primary intramuscular preadipocytes were isolated from longissimus dorsi muscles of 5-day-old Duroc-Landrace-Yorkshire piglets under sterile conditions [33]. The cells were cultured in high-glucose DMEM (Gibco, 11995) containing 10% fetal bovine serum (Gibco, 10091) and 1% penicillin-streptomycin. To induce adipogenic differentiation, 2 days post-confluent cells were cultured in induction medium containing 0.5 mM IBMX (Sigma Aldrich, I7018), 1 µM dexamethasone (Sigma-Aldrich, D1756) and 1 µg/mL insulin (Beyotime Biotechnology, P3376). After two days of differentiation, medium was replaced with a maintenance medium containing 1 µg/mL insulin and replaced every two days until lipid droplets were distinctly observed. All cells were maintained at 37 °C in a humidified 5% CO_2_ incubator. Cells were tested negative for mycoplasma contamination before use.

### Cell transfection

The transfection of small interfering RNA (siRNA) and plasmids were performed using Lipofectamine RNAiMAX (Invitrogen, 13778150) and Lipofectamine 2000 (Invitrogen, 11668019), respectively, according to the manufacturers’ instructions. All siRNA were ordered from Genepharma. The sequence for negative control siRNA is as follows (5’ to 3’): 5’-UUCUCCGAACGUGUCACGUTT-3’. Mouse *Ybx1* siRNA #1 and #2 targets 5ʹ-GUCAAAUGGUUCAAUGUAATT-3ʹ and 5ʹ-GGAGGCAGCAAAUGUUACATT-3ʹ, respectively. Mouse *Ulk1* and *Ulk2* siRNA targets 5ʹ-AAGGACCGCAUGGACUUUGAU-3ʹ and 5ʹ-CCAAAGACUCUGCGAGUAAUA-3ʹ, respectively. Porcine *Ybx1* siRNA #1 and #2 targets 5ʹ-GGAGUUUGAUGUUGUUGAATT-3ʹ and 5ʹ-GCAAGGCAGUAAAUAUGCATT-3ʹ, respectively. The YBX1- and enhanced green fluorescent protein (EGFP)-expressing adenovirus was generated from Hanbio. For adenoviral infection, 50% confluent cells were incubated with YBX1-or EGFP-expressing adenovirus in growth medium. The medium was then replaced and maintained in growth medium for 48 h.

### Oil Red O staining

Cells were washed and fixed in 4% paraformaldehyde at room temperature for 2 h. After washed with 60% isopropanol, the cells were stained with a filtered Oil Red O (Sigma-Aldrich, O1391) working solution for 15 min, then rinsed with distilled water and observed under a microscope. To quantify triglyceride accumulation, Oil Red O-stained lipids were eluted in 100% isopropanol, and the optical density was measured at 500 nm.

### Western blot analysis

Cells and tissues were lysed on ice in RIPA buffer with protease and phosphatase inhibitor cocktail (Beyotime Biotechnology, P1045) for 30 min. The protein concentration was determined using the BCA protein assay kit (Thermo Scientific, 23225). Protein samples were separated by SDS-PAGE and then transferred to polyvinylidene difluoride (Millipore, IPVH00010) membranes. The membranes were blocked with 5% non-fat milk at room temperature for 1 h and then incubated with corresponding primary antibodies overnight at 4 °C. Subsequently, the membranes were incubated with HRP-conjugated secondary antibodies (Biosharp, BL001A or BL003A) at room temperature for 1 h. Protein bands were visualized using FluorChem M Fluorescent Imaging System (ProteinSimple) and Light Chemiluminescence Kit (Epizyme, SQ201). The relative protein expression was calculated using the ImageJ software. Primary antibodies used in the experiment was listed in Supplementary Table S1. The original western blots were shown in Supplementary Material 2.

### Quantitative real-time PCR (qPCR) analysis

Total RNA from cells or adipose tissue were extracted using TRIzol reagent (Invitrogen, 15596018) and reverse transcribed into cDNA using reverse transcription kit (EZBioscience, A0010CGQ). qPCR analysis was performed using the SYBR Green qPCR Master Mix (EZBioscience, A0012-R2) with Applied Biosystems QuantStudio 3 Real-Time PCR System (Thermo Fisher Scientific). The data were analyzed following the 2^-ΔΔCt^ method and calculated using *Actb* as the normalization control. The primer sequences were listed in Supplementary Table 2.

### Immunofluorescence assay

For immunofluorescence assay, cells were fixed with 4% paraformaldehyde, permeabilized by 0.1% Triton X-100, and incubated with primary antibodies overnight. Then, the sample were staining with secondary antibody conjugated with FITC or Cy3 for 1 h. Nuclei were stained with DAPI for 5 min at room temperature. For staining of adipose tissue sections, samples were rapidly frozen using liquid nitrogen and fixed in Tissue-Tek OCT. Then the samples were sliced into 10 μm by a cryostat (Leica, CM1850) for staining. Immunofluorescent samples were imaged by confocal laser microscope (Carl Zeiss Ltd). To quantify the number of LC3 puncta, LC3 puncta with diameters between 0.3 μm and 1 μm were scored as positive.

### Transmission electron microscopy (TEM)

The cells were washed with PBS and fixed in 2.5% glutaraldehyde in phosphate buffer (0.1 M, pH 7.0) for 4 h and post-fixed with 1% OsO4 in phosphate buffer (0.1 M, pH 7.0) for 2 h. The samples were dehydrated with increasing concentrations of ethanol and an acetone series, then embedded in epoxy resin. Ultra-thin sections were cut and double-stained with uranyl acetate and lead citrate. Images were taken using JEM-2010 HR transmission electron microscopy (JEOL).

### Extraction of cytoplasmic and nuclear proteins

A nuclear and cytoplasmic protein extraction kit (Beyotime Biotechnology, P0028) was used according to the manufacturer’s instructions. Briefly, cells were harvested in cytoplasmic protein extraction buffer supplemented with protease and phosphatase inhibitor cocktail. The samples were incubated on ice for 15 min and centrifuged at 12,000 g for 5 min at 4 °C. The supernatant were collected as the cytoplasmic extracts. Next, the resulting pellet was resuspended in nuclear protein extraction buffer with protease and phosphatase inhibitor cocktail and incubated on ice for 30 min. The supernatant was collected as nuclear extracts following centrifuge at 12,000 g for 10 min.

### RNA Immunoprecipitation-qPCR (RIP-qPCR)

RIP-qPCR analysis was performed as described previously [34]. Briefly, FLAG-YBX1-overexpressing cells were lysed in lysis buffer (150 mM KCl, 10 mM HEPES, 2 mM EDTA, 0.5% NP-40, 0.5 mM DTT, protease inhibitor cocktail and RNase inhibitor) for 30 min at 4 °C. The lysates were centrifuged, and the supernatant was transferred to pass through a 0.22 μm membrane syringe. A small aliquot of lysate was saved as input, and the remaining sample was incubated with anti-FLAG or IgG magnetic beads (Beyotime Biotechnology, P2115 or P2171) for 4 h at 4 °C. Subsequently, the beads were washed with wash buffer (50 mM Tris, 200 mM NaCl, 2 mM EDTA, 0.05% NP40, 0.5 mM DTT, RNase inhibitor). Then the beads were eluted in wash buffer containing 3 × FLAG peptide (Beyotime Biotechnology, P9801). The input and immunoprecipitated RNAs were isolated by TRIzol and were reverse transcribed into cDNA. The fold enrichment was detected by qPCR. The RIP-qPCR primer sequences were listed in Supplementary Table 2.

### Cross-linking immunoprecipitation (CLIP)-qPCR

CLIP was performed as previously described [35]. Briefly, FLAG-YBX1-overexpressing cells were exposed to 0.15 J cm^−2^ ultraviolet light for crosslink. Then, cells were lysed and sonicated. A small aliquot of lysate was saved as input, and the remaining sample was incubated with anti-FLAG or IgG magnetic beads (Beyotime Biotechnology, P2115 or P2171) in RIP buffer (150 mM KCl, 25 mM Tris pH 7.4, 5 mM EDTA, 0.5 mM DTT, 0.5% IGEPAL® CA-630, 1× protease inhibitor) at 4 °C overnight. After washing with RIP buffer for three times, beads were resuspended in 80 µL PBS, followed by DNA digestion at 37 °C for 15 min and incubation with proteinase K at 37 °C for 15 min. Input and co-immunoprecipitated RNAs were recovered by TRIzol isolated and used for qPCR analysis. The primers were listed in Supplementary Table 2.

### Chromatin immunoprecipitation (ChIP)-qPCR

The ChIP-qPCR assay was performed using a ChIP Assay kit (Beyotime Biotechnology, P2078) according to the manufacturer’s instructions. Briefly, FLAG-YBX1-overexpressing cells were crosslinked with 1% formaldehyde in culture medium for 10 min at 37 °C followed by the addition of 125 mM glycine for 5 min. Cells were scraped into SDS lysis buffer supplemented with 1 mM PMSF. After that, crosslinked chromatin was shattered by sonication with 5 s on/45 s off for 10 cycles. Precleared chromatin samples were immunoprecipitated with FLAG antibody or control IgG at 4 °C overnight. 1% of the precleared chromatin samples lacking primary antibody was used as the Input. The association between YBX1 and target gene was detected by qPCR. The primers were designed based on promoter sequences and predicted site sequence on the JASPAR database and listed in Supplementary Table 2.

### Dual-luciferase reporter assay

The dual-luciferase reporter assay was performed as previously described [36]. The promoter of *Ulk1*-WT or *Ulk1*-MUT with mutant YBX1-binding site were inserted into pmirGLO Dual-Luciferase vector (Promega, E1330). The 5’UTR of *Ulk2*-WT or *Ulk2*-MUT (m^5^C was replaced by G) were amplified by PCR and were also inserted into pmirGLO Dual-Luciferase vector. For dual-luciferase reporter assay, cells seeded in 24-well plates were co-transfected with *Ulk1*-WT, *Ulk1*-MUT, *Ulk2*-WT or *Ulk2*-MUT and YBX1 siRNA or adenovirus. After 48 h post transfection, the activities of firefly luciferase and renilla luciferase in each well were determined by a Dual-Luciferase Reporter Assay System (Promega, E1910) according to the manufacturer’s protocol. Data were first normalized firefly luciferase activity to renilla luciferase activity.

### mRNA stability analysis

mRNA stability analysis was performed as described previously [36]. Briefly, Cells were treated with 5 µg/ml actinomycin D (Sigma-Aldrich, A9415) to inhibit global mRNA transcription. Samples were collected at the indicated time points. The total RNA was extracted for reverse transcription and the mRNA levels of interest were analyzed by qPCR. *Actb* was used as an internal control.

### Animals

C57BL/6 mice were housed under the controlled room temperature (22 °C ± 2 °C) with 12 h light and dark cycles and free access to water and food. For the HFD experiment, male mice at 4-week old were fed a high-fat diet containing 60% fat-derived calories (Research Diets, D12492) for 8 weeks. For adenoviral infection of inguinal WAT (iWAT), 8-week-old male mice fed with chow diet were first anesthetized using isoflurane. The iWAT was surgical exposed and injected with adenovirus. The incision was closed, and animals were allowed to recover. After 2 weeks of HFD feeding, the mice were sacrificed and iWAT were collected. For leupeptin treatment, 30 mg/kg of leupeptin was administered intraperitoneally 2 h before euthanasia. Serum leptin levels were measured by using Leptin ELISA kit according to the manufacturer’s instructions. For counting of adipocyte number, the whole iWAT were isolated from mice infected with Ad-EGFP or Ad-YBX1, and digested with 1 mg/ml type I collagenase (Gibco,17018029) at 37 °C for 30 min. Digested tissues were filtered through 100 μm cell strainers to remove large pieces, and the flow through were then centrifuged at 800 g for 10 min. The number of adipocytes in supernatant was measured by using a Cell Counting Kit-8 kit (Beyotime Biotechnology, C0037) according to the manufacturer’s instructions. All animal experiments were carried out according to the guidelines of The Animal Ethics Committee of South China Agricultural University.

### Statistical analysis

Data were presented as the mean ± SD from three independent experiments. Statistical significance was assessed by using a two-tailed student’s *t*-test or one‐way ANOVA followed by a Tukey test using GraphPad Prism software. Differences were considered statistically significant at *P < 0.05, **P < 0.01 and ***P < 0.001.

## Results

### Loss of YBX1 inhibits adipocyte differentiation and lipid accumulation of mouse 3T3-L1 and porcine preadipocytes

**Fig. 1:**
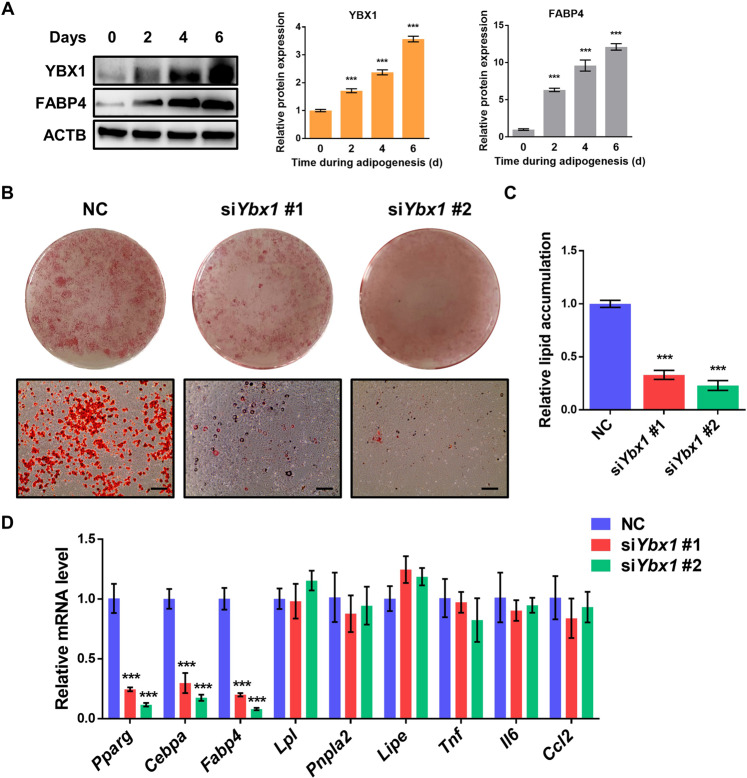
YBX1 deficiency attenuates adipogenesis and lipid accumulation of mouse 3T3-L1 preadipocytes. **A** Western blot analysis of YBX1 and FABP4 expression during adipogenesis of 3T3-L1 preadipocytes. **B** Oil Red O staining of negative control (NC) and YBX1 knockdown cells on day 6 of adipogenesis. Scale bar: 100 μm. **C** Relative lipid accumulation was quantified with a microplate spectrophotometer at 500 nm. **D** Real-time quantitative PCR (qPCR) analysis of *Pparg*, *Fabp4*, *Cebpa, Lpl, Pnpla2, Lipe, Tnf, Il6* and *Ccl2* expression in NC and YBX1 knockdown cells on day 6 of adipogenesis. The data were presented as the mean ± SD of triplicate tests. ***P < 0.001 compared to control group.

To investigate the function of YBX1 in adipogenesis, we first detected the expression profile of YBX1 during adipocyte differentiation of 3T3-L1 preadipocytes. The protein expression of YBX1 was gradually increased during differentiation, which was similar with adipocyte marker FABP4, suggesting YBX1 might be involved in the regulation of adipogenesis (Fig. 1A). Then, we performed loss-of-function assay using *Ybx1* siRNAs and confirmed the knockdown efficiency (Fig. S1A, B) by qPCR and western blot. Oil Red O staining analysis showed that silencing of YBX1 inhibited adipogenesis and decreased the accumulation of lipid droplets in 3T3-L1 cells (Fig. 1B,C). Moreover, the mRNA levels of the key adipogenic transcription factors and adipocyte markers peroxisome proliferator activated receptor gamma (*Pparg*), CCAAT/enhancer binding protein beta (*Cebpa*) and fatty acid binding protein 4 (*Fabp4*) were significantly suppressed in YBX1-deficient cells compared to control cells (Fig. 1D). However, silencing of YBX1 did not significantly affect the gene expression of lipolysis-related genes, such as lipoprotein lipase (*Lpl*), patatin-like phospholipase domain containing 2 (*Pnpla2/Atgl*) and lipase, hormone sensitive (*Lipe/Hsl*) (Fig. 1D), indicating that YBX1 didn’t affect lipolysis. Additionally, no significant difference was found in the gene expression of inflammatory cytokines, including tumor necrosis factor (*Tnf*), interleukin 6 (*Il6*) and chemokine (C-C motif) ligand 2 (*Ccl2*), between control and YBX1 deficiency cells (Fig. 1F). Furthermore, we found that knockdown of YBX1 in porcine preadipocytes also exhibited reduced lipid accumulation and decreased mRNA expression levels of adipogenic transcription factors and adipocyte markers when compared with control cells (Fig. S1A–E). These results demonstrate that YBX1 is required for adipocyte differentiation of mouse 3T3-L1 and porcine preadipocytes.

### YBX1 positively regulates adipogenesis by enhancing autophagy

**Fig. 2:**
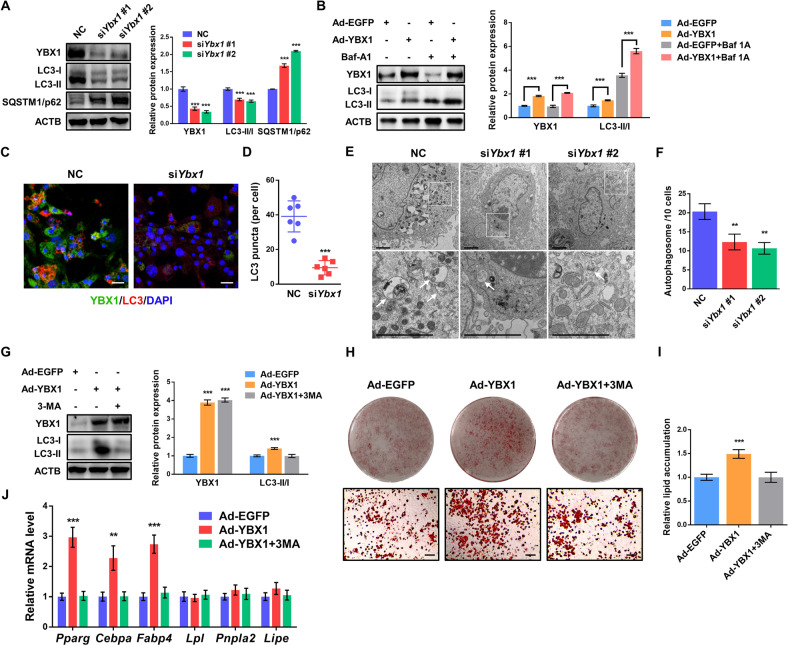
YBX1 facilities adipocyte differentiation through promoting autophagy. **A** Western blot analysis of YBX1, LC3 and SQSTM1 in NC and YBX1 knockdown 3T3-L1 cells. **B** Western blot analysis of YBX1 and LC3 in cells infected with EGFP- or YBX1-expressing adenovirus and treated with or without 10 nM Baf A1 for 4 h. **C** Immunofluorescence detection of YBX1 and LC3 in NC and YBX1 knockdown cells. Scale bar: 20 μm. **D** Statistical analysis of the number of LC3 puncta per cell. **E** Transmission electron microscope analysis of autophagosomes in NC and YBX1 knockdown cells. Arrows indicate autophagosomes. Scale bar: 2 μm. **F** Quantification of autophagosomes in cells. **G** Western blot analysis of LC3 in cells infected with EGFP- or YBX1-expressing adenovirus and treated with or without 10 mM 3-MA for 4 h. **H** Oil Red O staining of cells infected with EGFP- or YBX1-expressing adenovirus and induced to differentiate in the presence or absence of 5 mM 3-MA for 6 days. Scale bar: 100 μm. **I** Relative lipid accumulation was quantified with a microplate spectrophotometer at 500 nm. **J** qPCR analysis of *Pparg*, *Fabp4*, *Cebpa, Lpl, Pnpla2* and *Lipe* expression in cells infected with EGFP- or YBX1-expressing adenovirus and induced to differentiate in the presence or absence of 5 mM 3-MA for 6 days. The data were presented as the mean ± SD of triplicate tests. *P < 0.05, **P < 0.01, ***P < 0.001 compared to control group.

Numerous evidences have shown that autophagy plays an important role in regulating adipogenesis [37]. Given the recent studies reporting that YBX1 regulates autophagy in cancer cells and hepatic progenitor cells [23, 38], we then assessed the function of YBX1 in autophagy in adipocytes. We found that knockdown of YBX1 markedly downregulated the protein levels of microtubule-associated protein 1 light chain 3 beta (MAP1LC3B/LC3)-II:I ratio (an autophagy marker) and increased sequestosome 1 (SQSTM1/p62, a protein specifically degraded in lysosomes) compared to control cells (Fig. 2A). Furthermore, overexpression of YBX1 increased LC3-II:I ratio, which was furthered boosted when we treated 3T3-L1 cells with bafilomycin A_1_ (Baf A1) (Fig. 2B), a vacuolartype H + -translocating ATPase inhibitor that can block autophagosome-lysosome fusion [39]. Using immunofluorescence assay we observed a reduced amount of LC3 puncta were formed in YBX1-deficient cells (Fig. 2C,D). In addition, YBX1 deficiency attenuated autophagosome formation using transmission electron microscopy (TEM) (Fig. 2E,F). Consistent with 3T3-L1 cells, YBX1 deficiency decreased LC3-II:I ratio and inhibited autophagy activation in porcine adipocytes (Fig. S2A–E). These results indicate that YBX1 plays a conserved role in positively regulating autophagy among mouse and porcine preadipocytes.

To test whether YBX1 influences adipogenesis via activating autophagy, we overexpressed YBX1 and treated cells with or without autophagy inhibitor 3-methyladenine (3-MA) during adipogenesis. Forced expression of YBX1 enhanced autophagy, while 3-MA treatment could restore the enhanced autophagy by YBX1 overexpression (Fig. 2G). We found that 3-MA treatment recovered the promoted adipogenesis upon YBX1 overexpression (Fig. 2H,I). Consistently, the gene expression of *Pparg*, *Cebpa* and *Fabp4* were remarkably upregulated in YBX1-overexpressing cells, which could be reverse to normal level by 3-MA treatment (Fig. 2J). Overexpression of YBX1 did not significantly affect the gene expression of lipolysis-related genes (*Lpl*, *Pnpla2*, *Lipe*) (Fig. 2J). Collectively, these data reveal that YBX1 enhances adipogenesis through promoting autophagy.

### YBX1 deficiency reduces the expression of ULK1 and ULK2

**Fig. 3:**
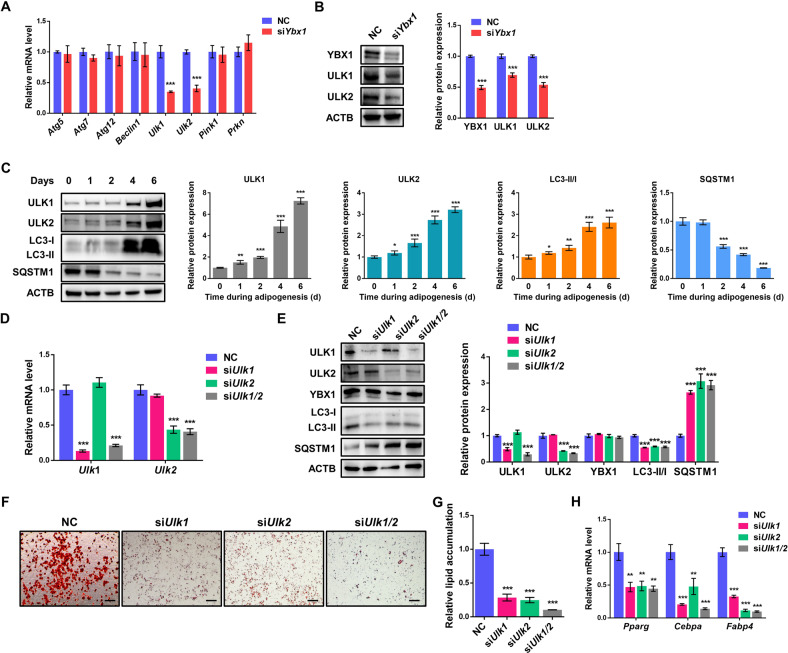
Loss of YBX1 decreases the expression of ULK1 and ULK2. **A** qPCR analysis of genes involved in autophagy in NC and YBX1 knockdown cells. **B** Western blot analysis of YBX1, ULK1 and ULK2 expression in NC and YBX1 knockdown cells. **C** Western blot analysis of ULK1, ULK2, LC3 and SQSTM1 expression during adipogenesis. **D** qPCR analysis of *Ulk1* and *Ulk2* in NC, ULK1 knockdown, ULK2 knockdown and ULK1/2 knockdown cells. **E** Western blot analysis of ULK1, ULK2, YBX1, LC3 and SQSTM1 in NC, ULK1 knockdown, ULK2 knockdown and ULK1/2 knockdown cells. **F** Oil Red O staining of NC, ULK1 knockdown, ULK2 knockdown and ULK1/2 knockdown cells on day 6 of differentiation. Scale bar: 100 μm. **G** Relative lipid accumulation was quantified with a microplate spectrophotometer at 500 nm. **H** qPCR analysis of *Pparg*, *Fabp4* and *Cebpa* expression in NC, ULK1 knockdown, ULK2 knockdown and ULK1/2 knockdown cells on day 6 of differentiation. The data were presented as the mean ± SD of triplicate tests. **P < 0.01, ***P < 0.001 compared to control group.

To dissect the key modulators governing YBX1-mediated autophagy, we tested the expression levels of autophagy-related genes upon YBX1 ablation. Recent studies reported that YBX1 could regulate autophagy through various autophagy-related genes in different cellular system, such as *Atg7*, Beclin 1 (*Becn1)*, PTEN induced kinase 1 (*Pink1)* and parkin RBR E3 ubiquitin protein ligase (*Prkn)* [23, 30, 38]. Intriguingly, our results demonstrated that YBX1 knockdown decreased the gene expression of *Ulk1* and *Ulk2*, while the levels of *Atg7*, *Becn1*, *Pink1*/*Prkn* and other autophagy-related genes were not significantly altered (Fig. 3A). Consistent with mRNA levels, depletion of YBX1 reduced the protein abundance of ULK1 and ULK2 (Fig. 3B). Previous studies showed that both of ULK1 and ULK2 played a role in regulating adipogenesis [8, 17, 19]. We measured the expression profile of ULK1 and ULK2 during adipogenesis and found that their protein levels were markedly increased at day 4 and 6 after differentiation (Fig. 3C), as similar with the expression pattern of YBX1. Moreover, the LC3 and SQSTM1 levels of 3T3-L1 cells were gradually increased and deceased, respectively, during adipogenesis (Fig. 3C), which was consistent with previous studies [19, 40, 41]. Consistently, knockdown of YBX1 deceased the gene and protein expression of ULK1 and ULK2 in porcine adipocytes (Fig. S3A, B). These findings indicate that *Ulk1* and *Ulk2* could be the target genes of YBX1 in our system.

To confirm the interaction between ULK1 and ULK2 and their functions on autophagy and adipogenesis, we knocked down ULK1 and ULK2, respectively or both, in 3T3-L1 cells. Silencing of ULK1 or ULK2 wouldn’t influence the gene and protein expression each other (Fig. 3D,E), indicating that YBX1 knockdown inhibited the expression of both ULK1 and ULK2. The protein abundance of YBX1 was unchanged upon knockdown of ULK1 or ULK2, proving the proposed upstream-downstream relationship between YBX1 and ULK1/2 (Fig. 3E). As expected, ablation of ULK1 and/or ULK2 decreased LC3 II:I ratio and increased SQSTM1 in 3T3-L1 cells (Fig. 3E), suggesting the repressed autophagy. Additionally, depletion of ULK1 and ULK2, respectively or both, markedly suppressed adipocyte differentiation and adipogenic gene expression compared to control cells (Fig. 3F–H). Together, these findings indicate that both of ULK1 and ULK2, as two potential targets of YBX1, are functionally significant for autophagy and adipogenesis.

### YBX 1 controls autophagy and adipogenesis by targeting both ULK1 and ULK2

**Fig. 4:**
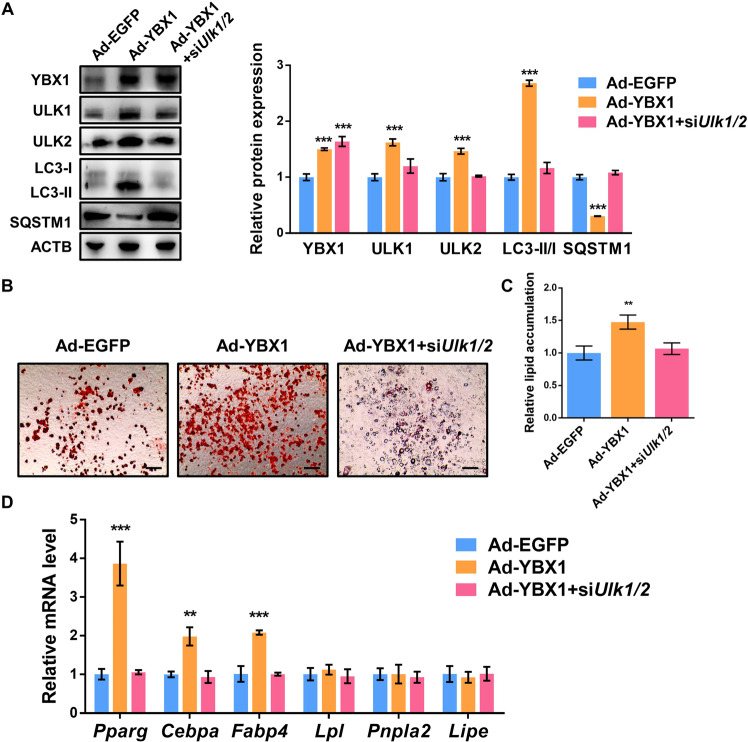
YBX1 regulates autophagy and adipogenesis by inhibiting both ULK1 and ULK2. **A** Western blot analysis of YBX1, ULK1, ULK2, LC3 and SQSTM1 in vector and YBX1-overexpressing cells transfected with or without *Ulk1*/*2* siRNA. **B** Oil Red O staining of vector and YBX1-overexpressing cells transfected with or without *Ulk1*/*2* siRNA on day 6 of adipogenesis. **C** Relative lipid accumulation was quantified with a microplate spectrophotometer at 500 nm. **D** qPCR analysis of *Pparg*, *Fabp4*, *Cebpa, Lpl, Pnpla2* and *Lipe* expression in vector and YBX1-overexpressing cells transfected with or without *Ulk1*/*2* siRNA. The data were presented as the mean ± SD of triplicate tests. **P < 0.01, ***P < 0.001 compared to control group.

To determine whether YBX1 regulates autophagy and adipogenesis through ULK1 and ULK2, we performed rescue experiment by knocking down ULK1 and ULK2 in YBX1-overexpressed 3T3-L1 cells. We found that YBX1 overexpression substantially increased ULK1 and ULK2 expression and LC3 II:I ratio, and decreased SQSTM1 expression when compared with control cells, while loss of ULK1 and ULK2 could recover the promoted LC3 II:I ratio and reduced SQSTM1 expression by YBX1 overexpression (Fig. 4A), indicating that YBX1 enhanced autophagy activation via mediating ULK1 and ULK2. Furthermore, depletion of ULK1 and ULK2 reversed the augmented adipogenesis and lipid accumulation in YBX1-overexpressed cells (Fig. 4B,C). Consistently, the upregulated gene expression of *Pparg*, *Cebpa* and *Fabp4* upon YBX1 overexpression were also restored (Fig. 4D), while the expression levels of lipolysis-related genes (*Lpl*, *Pnpla2*, *Lipe*) were unchanged (Fig. 4D). These data suggest that YBX1 promotes autophagy and adipogenesis through mediating both ULK1 and ULK2.

### YBX1 serves as an m^5^C binding protein promoting mRNA stability of *Ulk1*

**Fig. 5:**
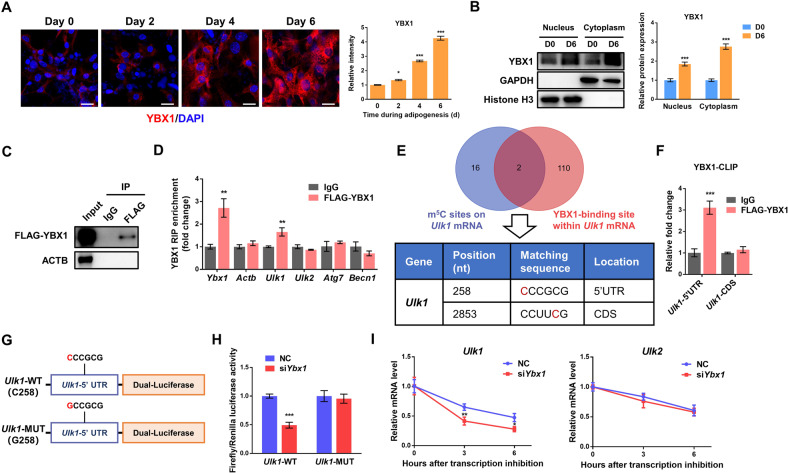
YBX1 as an m^5^C reader specifically binding and mediating mRNA stability of *Ulk1*. **A** Immunofluorescence detection of YBX1 during adipogenesis. Scale bar: 20 μm. **B** Western blot analysis of nuclear and cytoplasmic distribution of YBX1 in 3T3-L1 cells on day 0 or 6 of adipogenesis. Histone H3 and GAPDH serve as nuclear and cytoplasmic markers, respectively. **C** Western blot to confirm the immunoprecipitation of FLAG protein. **D** RNA immunoprecipitation-qPCR (RIP-qPCR) analysis of the interaction of *Ulk1* or *Ulk2* transcript with FLAG in cells overexpressing FLAG-tagged YBX1. *Ybx1* was used as a positive control, while *Actb* was used as a negative control. **E** Venn diagrams show 2 m^5^C sites are also predicted YBX1-binding sites within *Ulk1* transcripts by the RBPDB database. Red C in match sequence represents m^5^C. **F** RIP-qPCR analysis of the interaction of 5’UTR or CDS of *Ulk1* transcript with FLAG in cells overexpressing FLAG-tagged YBX1. **G** Schematic diagram of dual-luciferase reporter constructs containing a *Ulk1*-m^5^C wild type site (*Ulk1*-WT) or mutant site (*Ulk1*-MUT). **H** Relative luciferase activity of *Ulk1-*WT or *-*MUT luciferase reporter in NC and YBX1 knockdown cells. Firefly luciferase activity was measured and normalized to renilla luciferase activity. **I** mRNA lifetimes of *Ulk1* and *Ulk2* in NC and YBX1 knockdown cells. The data were presented as the mean ± SD of triplicate tests. *P < 0.05, **P < 0.01, ***P < 0.001 compared to control group.

YBX1 is identified as a multifunctional DNA/RNA binding protein that plays important roles in transcriptional and post-transcriptional regulation of gene expression [20]. To explore the underlying mechanism of YBX1 in regulating ULK1 and ULK2 expression, we first investigated the cellular distribution of YBX1 during adipogenesis. We found that both nuclear and cytoplasmic accumulation of YBX1 was increased at day 4 and 6 after differentiation (Fig. 5A), which was consistent with the upregulated expression of ULK1 and ULK2 (Fig. 3C). Also, we performed nuclear and cytoplasmic protein extraction and further validated the increased accumulation of YBX1 in both nucleus and cytoplasm after adipogenesis (Fig. 5B). These findings suggest that YBX1 might shuttle between cytoplasm and nucleus and bind to either mRNA or DNA of *Ulk1* and *Ulk2*.

To further investigate whether YBX1 recognizes *Ulk1* and *Ulk2* mRNAs in adipocytes, we infected the cells with YBX1-expressing adenovirus and performed RNA immunoprecipitation-qPCR (RIP-qPCR) with gene-specific primers. As shown in Fig. 5C, the FLAG-tagged YBX1 protein was successfully precipitated by using FLAG beads. The mature *Ybx1* mRNA was used as a positive control [42], *Actb* was chosen as the negative control. Interestingly, YBX1 was shown to interact with *Ulk1* mRNA, but not *Ulk2* mRNA (Fig. 5D). Furthermore, other autophagy genes such as *Atg7* and *Becn1* were not targets of YBX1 (Fig. 5D). Recent studies have identified YBX1 as an m^5^C modification “reader” protein recognizing m^5^C-modified mRNAs [25, 26]. Based on the published transcriptome bisulfite sequencing (BS-seq) data of mouse [43], *Ulk1* was identified to contain multiple m^5^C sites, suggest a possibility that YBX1 might bind *Ulk1* transcript through recognizing m^5^C modification. To test this hypothesis, we predicted YBX1 binding sites within *Ulk1* mRNA by using RBPDB database [44] and combined them with m^5^C sites. Two potential YBX1-binding m^5^C sites were found in 5’UTR and coding sequence (CDS), respectively, of *Ulk1* transcript (Fig. 5E). To confirm whether YBX1 binds these sites, we performed YBX1 crosslinking immunoprecipitation (CLIP)-qPCR assay with m^5^C site specific primers. YBX1 protein could directly interact with 5’UTR, but not CDS, of *Ulk1* mRNA (Fig. 5F). To determine the role of *Ulk1* m^5^C site in YBX1-regulated ULK1 expression, we constructed a pmirGLO plasmid containing the wile type 5’UTR fragment of *Ulk1* (*Ulk1*-WT) or 5’UTR with a mutated m^5^C site (*Ulk1*-MUT) and performed dual-luciferase reporter assay (Fig. 5G). YBX1 deficiency substantially reduced the luciferase activity of *Ulk1*-WT, but not *Ulk1*-MUT (Fig. 5H), providing further evidence that *Ulk1* expression required m^5^C modification at its 5′UTR specifically modulated by YBX1.

Next, we explored how YBX1 regulates *Ulk1* expression. Previous studies reported that YBX1 is involved in the regulation of mRNA stability, translation and splicing at the post-transcriptional level [20]. Since YBX1 positively regulated *Ulk1* mRNA expression, suggesting that YBX1 might mediate its mRNA stability. Using RNA stability assay, we observed a significantly shortened *Ulk1* mRNA half-life by YBX1 knockdown, while the mRNA stability of *Ulk2* was unchanged (Fig. 5I). Taken together, these results demonstrate that YBX1 targets and stabilizes mRNA of *Ulk1*, but not *Ulk2*, as an m^5^C binding protein in 3T3-L1 adipocytes.

### YBX1 regulates *Ulk2* expression through promoting its transcription

**Fig. 6:**
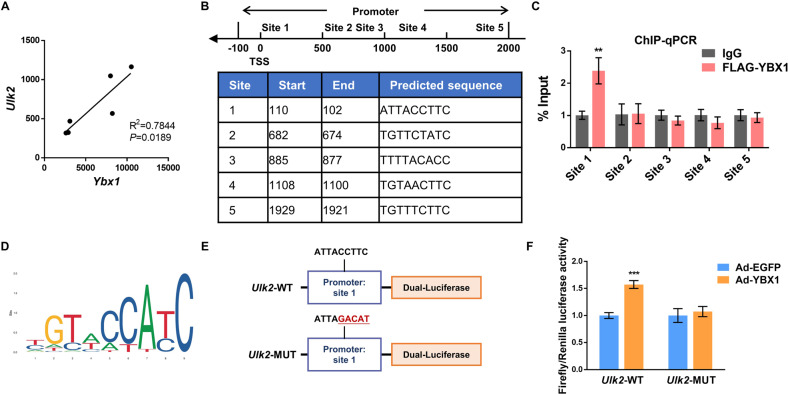
Knockdown of YBX1 reduces *Ulk2* expression *via* inhibiting its transcription. **A** Pearson’s correlation analysis of the relative expression levels of *Ybx1* and *Ulk2* in 3T3-L1 adipocytes by using published RNA-seq data (GSE135683) from GEO datasets. **B** Putative YBX1-binding site(s) within promoter of *Ulk2* were predicted by using JASPAR database. TSS, transcription start site. **C** ChIP-qPCR analysis of the association of YBX1 with the promoter of *Ulk1* and *Ulk2* in 3T3-L1 cells. Nonspecific IgG was used as a negative control. **D** Consensus YBX1 binding sites were obtained from JASPAR database. **E** Schematic diagram of dual-luciferase reporter constructs containing a YBX1-binding wild type (*Ulk2-*WT) or mutated (*Ulk2-*MUT) site within *Ulk2* promoter. **F** Relative luciferase activity of *Ulk2-*WT or -MUT luciferase reporter in vector and YBX1 overexpressing cells. Firefly luciferase activity was measured and normalized to renilla luciferase activity. The data were presented as the mean ± SD of triplicate tests. **P < 0.01, ***P < 0.001 compared to control group.

Since YBX1 doesn’t regulate *Ulk2* expression at the post-transcriptional level, it is highly possible that YBX1 promotes *Ulk2* transcription as a transcription factor. We first conducted correlation analysis of published RNA profile data of 3T3-L1 cells from GEO database (GSE135683), and confirmed that the relative expression of *Ybx1* was positively associated with *Ulk2* mRNA level in adipocytes (R^2^ = 0.7844, *P* < 0.05) (Fig. 6A). We next investigated the underlying mechanism of YBX1 in regulating *Ulk2* expression. Five potential YBX1 binding sites were predicted within 2000 bases upstream and 100 bases downstream of the *Ulk2* transcription start site by using JASPAR database (Fig. 6B). Using chromatin immunoprecipitation-qPCR (ChIP-qPCR) assay, we found that YBX1 had a significant enrichment on site 1 over IgG control (Fig. 6C), indicating a direct binding of YBX1 with *Ulk2* promotor. To further validate YBX1 regulates *Ulk2* expression at the transcriptional level, we performed luciferase reporter assays by inserting the wile type or mutated site 1 sequence of *Ulk2* promoter into reporter plasmid (Fig. 6D). We found that overexpression of YBX1 promoted the luciferase activity of *Ulk2*-WT, but not *Ulk2*-MUT. Collectively, these results suggest that YBX1 modulates *Ulk2* expression via promoting its transcription.

### YBX1 overexpression enhances autophagy and adipose tissue expansion in vivo

**Fig. 7:**
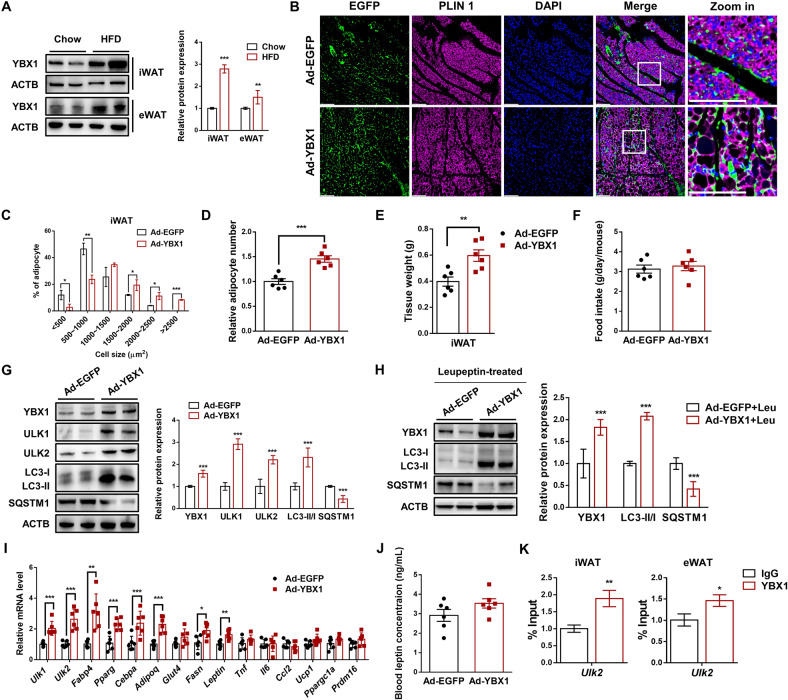
YBX1 regulates autophagy and adipose tissue expansion in vivo. **A** Western blot analysis of the protein expression of YBX1 in iWAT and eWAT from male mice fed a chow diet or HFD for 8 weeks. **B** Immunofluorescence detection of EGFP and PLIN1 in iWAT from mice infected with Ad-EGFP or Ad-YBX1 and fed a HFD for 2 weeks. Nuclei are stained with DAPI. Scale bar, 100 μm. **C** Adipocyte sizes of iWAT from mice infected with Ad-EGFP or Ad-YBX1 and fed a HFD for 2 weeks. **D** Adipocyte number of iWAT from mice infected with Ad-EGFP or Ad-YBX1 and fed a HFD for 2 weeks. **E** Tissue weight of iWAT from mice infected with Ad-EGFP or Ad-YBX1 and fed a HFD for 2 weeks. **F** Food intake of mice infected with Ad-EGFP or Ad-YBX1 and fed a HFD for 2 weeks. **G** Western blot analysis of YBX1, ULK1, ULK2, LC3, and SQSTM1 in iWAT from mice infected with Ad-EGFP or Ad-YBX1 and fed a HFD for 2 weeks. **H** Western blot analysis of YBX1, LC3, and SQSTM1 in iWAT from mice infected with Ad-EGFP or Ad-YBX1 and treated with leupeptin. **I** qPCR analysis of gene expression of *Ulk1*, *Ulk2*, adipogenesis markers, inflammatory cytokines and browning-related genes in iWAT from mice infected with Ad-EGFP or Ad-YBX1 and fed a HFD for 2 weeks. **J** Plasma leptin levels were determined by ELISA on blood samples obtained from mice infected with Ad-EGFP or Ad-YBX1. **K** ChIP-qPCR analysis of the association of YBX1 with the promoter of *Ulk1* and *Ulk2* in iWAT and eWAT from mice infected with Ad-EGFP or Ad-YBX1. Nonspecific IgG was used as a negative control. The data were presented as the mean ± SD of triplicate tests. *P < 0.05, **P < 0.01, ***P < 0.001 compared to control group.

To investigate the role of YBX1 in white adipose tissue in vivo, we first detected the protein abundance of YBX1 in WAT of mice fed with chow diet or HFD. As shown in Fig. 7A, YBX1 expression in inguinal WAT (iWAT) and epididymal WAT (eWAT) were highly induced by HFD compared with chow diet group, implying a regulatory role of YBX1 in white adipose tissue expansion. Next, we specifically injected EGFP-or YBX1-expressing adenovirus into iWAT of 8-week-old mice. After 2 weeks of HFD feeding, we found that iWAT from mice infected with Ad-EGFP or Ad-YBX1 robustly expressed EGFP, indicating the success of virus infection and YBX1 overexpression (Fig. 7B). Compared with control mice, mice infected with Ad-YBX1 contain larger lipid droplets in iWAT determined by lipid droplet-associated protein perilipin 1 (PLIN1) (Fig. 7B). Consistently, mice infected with Ad-YBX1 exhibited larger size of adipocytes in iWAT than control group (Fig. 7C). Furthermore, YBX1 overexpression in iWAT increased the number of mature adipocytes in iWAT (Fig. 7D). As expected, overexpression of YBX1 significantly increased iWAT mass when compared with control group (Fig. 7E). No significant difference was found in food intake between control and YBX-overexpressed mice (Fig. 7F). These results demonstrate that overexpression of YBX1 promotes adipose tissue expansion in vivo.

To test whether YBX1 influences adipose tissue expansion through ULK1/ULK2-mediated autophagy in vivo, we detected the LC3-II:I ratio and ULK1/2 expression in iWAT from mice infected with Ad-EGFP or Ad-YBX1. Forced expression of YBX1 significantly increased LC3-II:I ratio and decreased SQSTM1 expression in iWAT (Fig. 7G). To further validate the role of YBX1 on autophagy in vivo, we treated mice with protease inhibitor leupeptin and found that YBX1 overexpression enhanced LC3-II formation (Fig. 7H), suggesting the enhanced autophagy. Furthermore, the protein expression of ULK1 and ULK2 were elevated upon YBX1 overexpression (Fig. 7G). We also found that the mRNA levels of *Ulk1* and *Ulk2* were upregulated in iWAT from mice infected with Ad-YBX1 when compared with control group (Fig. 7H), indicating that YBX1 promoted autophagy through enhancing ULK1 and ULK2 as shown in cell study. Consistent with the phenotype, YBX1 overexpression markedly increased the gene expression of adipogenesis markers, such as *Fabp4*, *Pparg*, *Cebpa*, adiponectin (*Adipoq*), fatty acid synthase (*Fasn*) and *Leptin* in iWAT (Fig. 7I). Since it has been proposed that leptin may regulate autophagy in vivo. To test whether YBX1 overexpression increased plasma leptin concentrations, which in turn promoted autophagy in WAT, we measured the blood leptin levels of mice. No difference was found in plasma leptin levels between mice infected with Ad-EGFP or Ad-YBX1 (Fig. 7J), indicating that YBX1 overexpression directly promoted autophagy, then enhanced adipose tissue expansion and expression of *Leptin*. In addition, overexpression of YBX1 in iWAT didn’t influence the mRNA levels of inflammatory cytokines, such as *Tnf, Il6* and *Ccl2* (Fig. 7I). A previous study showed the potential role of YBX1 on promoting browning of white adipocytes in vitro [29]. Therefore, we detected the effect of YBX1 overexpression on the expression levels of browning-related genes in iWAT and found that the gene expression of uncoupling protein 1 (*Ucp1*), PPARG coactivator 1 alpha (*Ppargc1a*) and PR domain containing 16 (*Prdm16*) was unchanged after 2 weeks of YBX1 overexpression and HFD feeding at room temperature when compared with control group (Fig. 7I). Furthermore, we performed ChIP-qPCR assay and confirmed that YBX1 could bind to promoter of *Ulk2* in iWAT and eWAT (Fig. 7K). Taken together, these results suggest that YBX1 enhances autophagy through facilitating ULK1 and ULK2 expression, thereby promoting adipose tissue expansion in vivo.

## Discussion

YBX1 is a well-established oncogene and a multifunctional protein, which regulates cell proliferation, differentiation, autophagy, apoptosis and stress response [45]. Emerging evidences showed that YBX1 expression was upregulated in brown adipose tissue (BAT) and subcutaneous WAT from mice upon cold exposure and β-adrenergic agonist treatment [29, 30]. YBX1 drove the thermogenic fate of adipocytes at the transcriptional level [29]. Our recent study found that YBX1 served as an RNA-binding protein regulating brown adipogenesis in vitro and thermogenesis of BAT in vivo [30]. However, the role and function of YBX1 in white adipogenesis is still unclear. In this study, we performed loss- and gain-of-function experiments and found that YBX1 was required for white adipogenesis and lipid accumulation, which is conserved among murine and porcine adipocyte. Our in vivo study showed that overexpression of YBX1 in iWAT promoted adipose tissue expansion, which was driven by the increase in number (hyperplasia) and size (hypertrophy) of adipocytes. Although inflammation are often associated to adipocyte hypertrophy, no significant difference was found in the gene expression of inflammatory cytokines in iWAT between control and YBX1-overexpression group. This could be explained by 2-week YBX1 overexpression might be not enough to induce inflammation. Additionally, we found that 2-week YBX1 overexpression in iWAT increased the expression of adipogenesis markers, but not browning-related genes. Since beige adipocyte biogenesis can be induced by chronic cold exposure or β3-AR agonists [46], but inhibited by HFD [47], the effect of YBX1 on browning of WAT could be undetectable under HFD and room temperature condition. Collectively, these findings suggest that YBX1 plays a distinct role in different fat depots and adipocytes, which could be a potential target for treatment of obesity. WAT-specific YBX1 knockout (such as *Adipoq*-cre and *Ybx1* floxed mice) or overexpression mice would be needed for further investigate the physiologic role of YBX1 in WAT. Additionally, stromal vascular fraction (SVF) cells from iWAT and eWAT can be isolated, respectively, to investigate whether YBX1 plays a distinct role in regulation of iWAT and eWAT in vitro.

Emerging evidences have shown that YBX1 was involved in the regulation of autophagy in diverse cell types. A recent study showed that the protein expression of YBX1 was positively correlated with autophagy level in clinical lung tumor tissue [23]. YBX1 promoted autophagy and decreased drug sensitivity of human non-small cell lung cancer cells by targeting p110β/Vps34/BECN1 pathway. Another study reported that long noncoding RNA Linc00857 interacting with YBX1 to regulate autophagy and proliferation of lung cancer cells through MET proto-oncogene (MET)/AMPK signaling [48]. Linc02527 directly binding to YBX1 and activated CDKN1A, then the autophagy was promoted in human trophoblasts [24]. In mice, YBX1 facilitated proliferation of hepatic progenitor cells and liver fibrogenesis through transcriptionally regulating ATG7-mediated autophagy [38]. Our previous work showed that YBX1 promoted mitophagy via increasing *Pink1* and *Prkn* expression, thereby enhancing brown adipogenesis and thermogenesis in mice [30]. In the current study, we found that YBX1 promoted autophagy in white adipocyte through upregulating the expression of *Ulk1* and *Ulk2*. The gene expression of other autophagy-related genes such as *Becn1*, *Atg7*, *Pink1*, *Prkn* and so on, were unchanged. Consistent with in vitro data, YBX1 overexpression enhanced ULK1/ULK2 expression and autophagy in iWAT. Of note, we cannot rule out the possibility that YBX1 regulate autophagy-related regulators or pathway. These results indicate that the positive correlation between YBX1 and autophagy is conserved in diverse cell types across species, however, the molecular mechanism of YBX1 in regulating autophagy could be cell-specific.

In the present study, we identified ULK1 and ULK2 as key regulators in YBX1-mediated autophagy and adipogenesis in adipocytes. ULK1 and ULK2 participate in forming the early membrane structure of autophagosomes and play a key role in regulating the induction of autophagy [49]. We found that the expression levels of ULK1 and ULK2 were largely increased in 3T3-L1 cells after differentiation, implying that ULK1 and ULK2 have important functions in differentiating or differentiated adipocytes. ULK1 and ULK2 were previously reported to had distinct functions in the regulation of adipogenesis [8]. ULK2 inhibition suppressed autophagy and adipogenesis. However, ULK1 knockdown didn’t affect adipogenesis, because loss of ULK1 reduced fatty acid oxidation and lipid accumulation of adipocytes. In contrast, we found ULK1 and ULK2 were both essential for adipocyte differentiation. This discrepancy could potentially be explained by the dual role and function of ULK1 at the early stage or late stage of adipogenesis. For example, a recent study found that the inhibitory phosphorylation of ULK1 induced by SIRT1 attenuated autophagy, leading to suppressed differentiation of 3T3-L1 preadipocyte [19]. AMPK positively regulated autophagy by increasing ULK1 activity, thereby promoting adipogenesis of ischemia-challenged adipose-derived stem cells [18]. Estradiol signaling inhibited ULK1, thereby suppressing autophagy and adipogenesis [17], suggesting a pro-adipogenic role of ULK1 at the early stage of adipogenesis, which is consistent with our results. Other studies revealed a distinct role of ULK1 in differentiated adipocytes. In mature adipocytes, SIRT3 activated autophagy on lipid droplets (LDs) through activating ULK1, leading to smaller LDs and decreased lipid accumulation [50]. Sulforaphane was recently reported to induced lipophagy and decreased the size of LDs in differentiated 3T3-L1 adipocytes through activation of AMPK-mTOR-ULK1 pathway [51]. Whether ULK2 has multiple functions in adipocyte should be further investigated.

YBX1 is a nuclear–cytoplasmic shuttling and multifunctional protein and regulates gene expression at the post-transcriptional and transcriptional levels [20, 21]. In YBX1, the CSD plays a key role in the interactions with both DNA and RNA and is highly homologous among different organisms [52]. In the present study, we found that the YBX1 expression is increased in the cytoplasm and nucleus during adipogenesis, implying a dual role of YBX1 in regulating *Ulk1* and *Ulk2* expression. For transcriptional function, YBX1 were first identified as a DNA-binding protein involved in regulating transcription of many genes in various organisms and were thought to specifically bind to DNAs containing the so-called Y-box motif [20]. However, later studies demonstrated that YBX1 change the expression of genes both with and without the Y-box sequence in their promoters. Previous study showed that of YBX1 deficiency inhibited white adipocytes browning partially through mediating histone demethylase jumonji domain containing 1 C (JMJD1C) at transcriptional level [29]. Using ChIP-qPCR and dual-luciferase reporter assay, we identified YBX1 bond to the promoter of *Ulk2* and promoted its transcription and expression. Nevertheless, we cannot exclude the possibility of YBX1 binding to the promoter of other autophagy-associated genes under specific circumstance, which will be further studied by using ChIP-seq in the future. Numerous studies found that YBX1 as an RNA-binding protein regulating mRNA stability, splicing, packaging and translation [21]. YBX1 can interact with any RNA sequence, although A- and C-rich sites are somewhat more preferable. Our recent work demonstrated that YBX1 facilitated mitophagy and brown adipogenesis via promoting mRNA stability of *Pink1* and *Prkn* [30]. Recent studies unveiled that YBX1 was a novel m^5^C reader recognizing m^5^C-modified mRNAs through the indole ring in its CSD [25]. YBX1 targeted oncogene heparin binding growth factor (*HDGF*) m^5^C and stabilize its mRNA to promote pathogenesis of bladder cancer. Intriguingly, *Ulk1* was identified to contain an m^5^C site in its 5’UTR and CDS [43]. Using RIP-qPCR and CLIP-qPCR, we proved that YBX1 bond to 5’UTR of *Ulk1* mRNA through recognizing m^5^C modification. The 5’UTR is the primary targets for mRNA degradation pathways, and play critical roles in mRNA stability [53]. The CSD of YBX1 is responsible for the mRNA stabilizing activity [54]. We found that YBX1 increased ULK1 expression in an m^5^C-dependent manner through promoting its mRNA stability. Since the prevalence distribution of m^5^C on mRNAs, whether YBX1 regulates autophagy and adipogenesis via targeting other transcripts should be further determined by RIP -seq and BS-seq. A recent study reported a role of YBX1 in interacting with the readers of *N*^6^-methyladenosine (m^6^A), another RNA modification, and stabilizing m^6^A-modified RNA for maintaining myeloid leukemia cell survival [55]. YBX1 acted as either a stabilizer or a scaffold for recruiting other proteins, suggesting that it might affect mRNA fates in multiple ways. Given the diverse roles of YBX1 in regulating transcription and post-transcription, YBX1 may act in distinct manners on transcripts in different contexts, which needs to be further investigated.

**Fig. 8:**
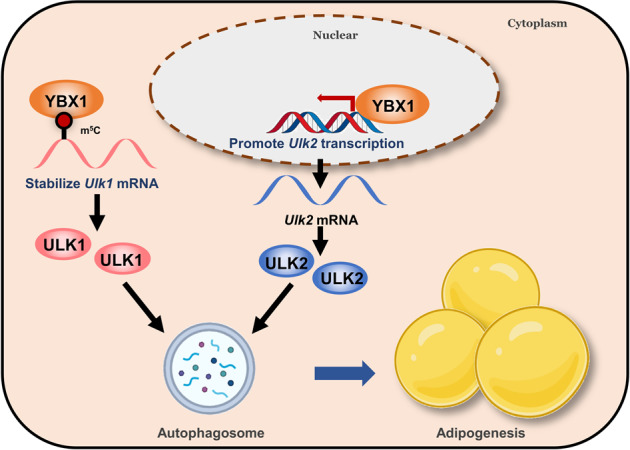
Proposed model depicting the role and regulation of YBX1 in autophagy and adipogenesis. YBX1 specifically binds to m^5^C-containing *Ulk1* transcript and stabilizes its mRNA as an RNA-binding protein. Meanwhile, YBX1 serves as a DNA-binding protein promoting *Ulk2* transcription, leading to increased protein abundance of ULK1 and ULK2, thereby enhancing autophagy and adipogenesis.

In summary, our findings provide novel insights into the underlying molecular mechanisms of YBX1 in modulation of ULK1/ULK2 expression at the post-transcriptional and transcriptional level in adipocytes (Fig. 8). This work unveils the functional significance of YBX1 in regulating autophagy and adipogenesis, suggesting a potential therapeutic strategy for treatment of obesity and metabolic diseases.

## Supplementary information

Supplementary Material 1

Supplementary Material 2

Figure S1

Figure S2

Figure S3

check list

## Data Availability

The data that support the findings of this study are available in the methods and supplementary material of this article. Additional data are available from the corresponding author on reasonable request.
