# Supplementary material for: Transcriptional and post-transcriptional control of autophagy and adipogenesis by YBX1
Source: Cell Death Dis. 2023 Jan 16;14(1):29. doi: 10.1038/s41419-023-05564-y (PMC9841012; doi:10.1038/s41419-023-05564-y)
Supplement: Supplementary file 1 — Supplementary Material 1 [file 41419_2023_5564_MOESM1_ESM.docx]

**Supplementary Material 1**

Supplementary Figures and Tables

**Fig. S1: YBX1 deficiency inhibits adipogenesis of porcine primary preadipocytes.**

**A, B** YBX1 knockdown efficiency in 3T3-L1 preadipocytes was measured by qPCR (**A**) and western blot (**B**). **C, D** YBX1 knockdown efficiency in porcine preadipocytes was measured by qPCR (**C**) and western blot (**D**). **E** Oil Red O staining of negative control (NC) and YBX1 knockdown porcine adipocytes on day 8 of adipogenesis. Scale bar: 100 μm. **F** Relative lipid accumulation was quantified with a microplate spectrophotometer at 500 nm. **G** Real-time quantitative PCR (qPCR) analysis of *PPARG*, *FABP4* and *CEBPA* expression in NC and YBX1 knockdown porcine adipocytes on day 8 of adipogenesis. The data were presented as the mean ± SD of triplicate tests. ^**^*P* < 0.01, ^***^*P* < 0.001 compared to control group.

**Fig. S2: Loss of YBX1 inhibits autophagy in porcine adipocytes.**

**A** Western blot analysis of LC3 and SQSTM1 in NC and YBX1 knockdown porcine adipocytes. **B** Immunofluorescence assays of LC3 in NC and YBX1 knockdown porcine adipocytes. Scale bar: 20 μm. **C** Statistical analysis of the number of LC3 puncta per cell. **D** Transmission electron microscope analysis of autophagosomes in NC and YBX1 knockdown porcine adipocytes. Arrows indicate autophagosomes. Scale bar: 1 μm. **E** Quantification of autophagosomes in cells. The data were presented as the mean ± SD of triplicate tests. ^**^*P* < 0.01, ^***^*P* < 0.001 compared to control group.

**Fig. S3: Knockdown of YBX1 decreases the expression of ULK1 and ULK2 in porcine adipocytes**.

**A** qPCR analysis of expression levels of *ULK1* and *ULK2* in control and YBX1 knockdown porcine preadipocytes. **B** Western blotting analysis of ULK1 and ULK2 in control and YBX1 knockdown porcine preadipocytes. The data were presented as the mean ± SD of triplicate tests. ^***^*P* < 0.001 compared to control group.

**Supplementary Tables**

**Supplementary Table 1. The information of antibodies used in this study.**

| **Antibodies** | **Source** | **Identifier** | **Host** |
| --- | --- | --- | --- |
| ACTB | Bioworld | AP0060 | Rabbit |
| FLAG | Huabio | 0912-1 | Rabbit |
| GAPDH | Bioworld | P04406 | Rabbit |
| Histone H3 | Cell Signaling Technology | 4499 | Rabbit |
| MAP1LC3B | ABclonal | A19665 | Rabbit |
|  | Cell Signaling Technology | 83506 | Mouse |
| SQSTM1/p62 | Cell Signaling Technology | 8025 | Rabbit |
| ULK1 | Huabio | ET1704-63 | Rabbit |
| ULK2 | ABclonal | A15244 | Rabbit |
| YBX1 | Proteintech | 20339-1-AP | Rabbit |

**Supplementary Table 2. Sequences of primers.**

| **Gene** | **Species** | **Forward primer (5’-3’)** | **Reverse primer (5’-3’)** |
| --- | --- | --- | --- |
| *Actb* | mouse | TGAGCTGCGTTTTACACCCT | GCCTTCACCGTTCCAGTTTT |
| *Adipoq* | mouse | GTTCCCAATGTACCCATTCGC | TGTTGCAGTAGAACTTGCCAG |
| *Atg5* | mouse | TGTGCTTCGAGATGTGTGGTT | GTCAAATAGCTGACTCTTGGCAA |
| *Atg7* | mouse | GCTGCTGAGATCTGGGACAT | GAGATGTGGAGATCAGGACCAG |
| *Atg12* | mouse | CACACATGGCAGCACTCCTA | TTCCCCCAGAGGTGAGACAA |
| *Becn1* | mouse | ATGCAGGTGAGCTTCGTGTG | AATGGCTCCTGTGAGTTCCTG |
| *Ccl2* | mouse | CTGTGCTGACCCCAAGAAGG | AAGACCTTAGGGCAGATGCAG |
| *Cebpa* | mouse | GGTTTCGGGTCGCTGGATCTCTAG | ACGGCCTGACTCCCTCATCTTAGAC |
| *Fabp4* | mouse | CCTTCAAACTGGGCGTGGAA | TCCATCCCACTTCTGCACCT |
| *Fasn* | mouse | GGAGGTGGTGATAGCCGGTAT | TGGGTAATCCATAGAGCCCAG |
| *Glut4* | mouse | TGTCACAGGAGAAGCAGGTG | GCTCCAATCGTGGCATAGAT |
| *Lpl* | mouse | AAGCCGTCATTGCTGGTG | GCTGGATGGTTTGCGTTCT |
| *Il6* | mouse | AGTTGCCTTCTTGGGACTGA | CCTCCGACTTGTGAAGTGGT |
| *Lipe* | mouse | GGGAGCTCCAGTCGGAAGA | GTCTTCTGCGAGTGTCACCA |
| *Pnpla2* | mouse | CTGAGAATCACCATTCCCACATC | CACAGCATGTAAGGGGGAGA |
| *Pparg* | mouse | TGGGTGAAACTCTGGGAGATTC | AGAGGTCCACAGAGCTGATTCC |
| *Ppargc1a* | mouse | TTGACTGGCGTCATTCGGG | CACCACTTCAATCCACCCAGA |
| *Prdm16* | mouse | CAGCACGGTGAAGCCATTC | GCGTGCATCCGCTTGTG |
| *Tnf* | mouse | ACGGCATGGATCTCAAAGAC | GTGGGTGAGGAGCACGTAGT |
| *Ucp1* | mouse | ACTGCCACACCTCCAGTCATT | CTTTGCCTCACTCAGGATTGG |
| *Ulk1* | mouse | GCAAGTTCGAGTTCTCTCGC | GTCGTGCTTCTCGCGGT |
| *Ulk2* | mouse | CCATGAGAAGCGACCGGG | GATGGACACTCATGCCGAGA |
| *Ybx1* | mouse | GCAGACCGTAACCATTATAGACG | TCTCCGCATGTAGTAAGGTGG |
| *CEBPA* | pig | GGTTTCGGGTCGCTGGATCTCTAG | ACGGCCTGACTCCCTCATCTTAGAC |
| *FABP4* | pig | GACGACAGGAAGGTGAAGAG | ACATTCCACCACCAGCTTGT |
| *GAPDH* | pig | ACACTCACTCTTCTACCTTTG | GAAATTCATTGTCGTACCAG |
| *PPARG* | pig | AGGACTACCAAAGTGCCATCAAA | GAGGCTTTATCCCCACAGACAC |
| *ULK1* | pig | GGACCCCATCTCCTTCCCC | TGCTCAATGCGCTGGTAGTT |
| *ULK2* | pig | TGGGGCCTGTGTCTGCT | GAACACGCGGATGGTGTCT |
| *YBX1* | pig | AGACAAAAGCAGCCGATCCA | GGTGGATGACCGGATGATGG |
| **RIP-qPCR primers** | | | |
| *Ulk1* 5’UTR | mouse | TCAGGCCTGCCCGATGG | TCGAACTTGCCCACGGTCTC |
| *Ulk1* CDS | mouse | ACTGCTGTAGCCTTGCTGAC | TGTGCTCTTGCTCCATGAGG |
| **RIP-qPCR primers** | | |  |
| *Ulk2* site 1 | mouse | CCTCTTGAGTTAGACAAAGACACT | TCCCTCCTATGCAACCTAGAC |
| *Ulk2* site 2 | mouse | AAGTAGAGGGCATGTGGCAG | TCACCAGCTCCAGTTTTCCC |
| *Ulk2* site 3 | mouse | CTTCTGGTTTGGTTTGGTCCAG | GAGTCCTAAAGGCCCCAAGATA |
| *Ulk2* site 4 | mouse | AGCCAAGTGTGGCAGTGATTAT | GTGCTCACACTCTTGTCTAGGAA |
| *Ulk2* site 5 | mouse | AACCTAGGGCTTCACAGTTCC | CAGAGCAAGATCATCTCAATAACCC |
