# Supplementary material for: Transcriptional and post-transcriptional control of autophagy and adipogenesis by YBX1
Source: Cell Death Dis. 2023 Jan 16;14(1):29. doi: 10.1038/s41419-023-05564-y (PMC9841012; doi:10.1038/s41419-023-05564-y)

Supplementary Material 2

Original western blots

Fig. 1A

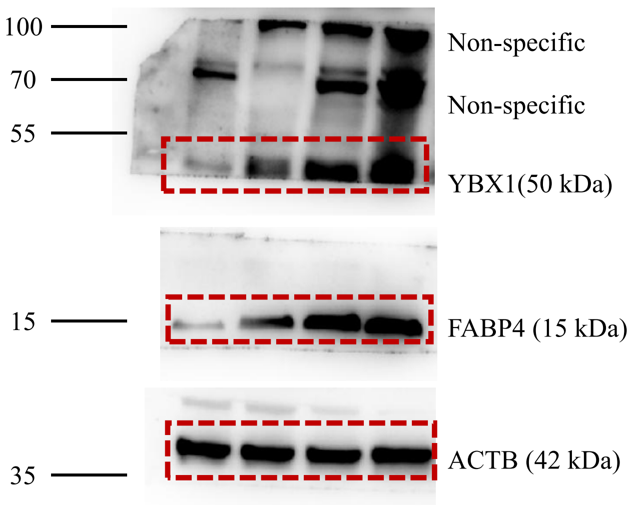

Fig. 2A

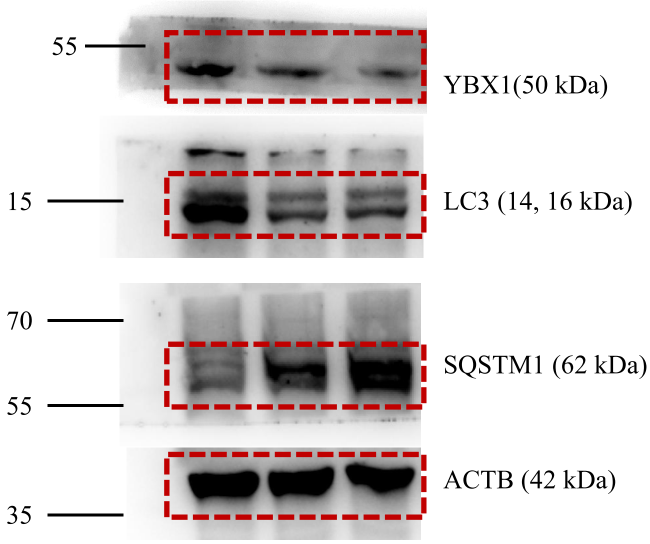

Fig. 2B

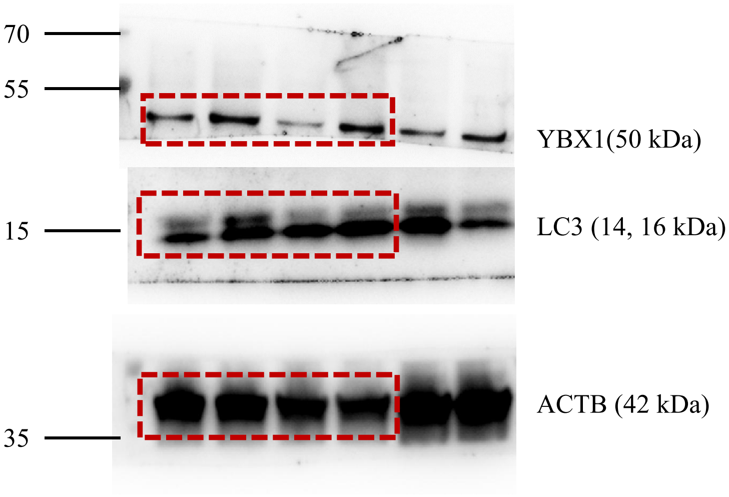

Fig. 2G

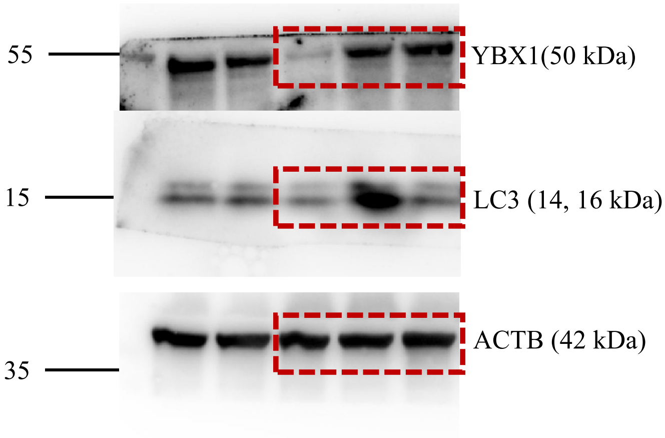

Fig. 3B

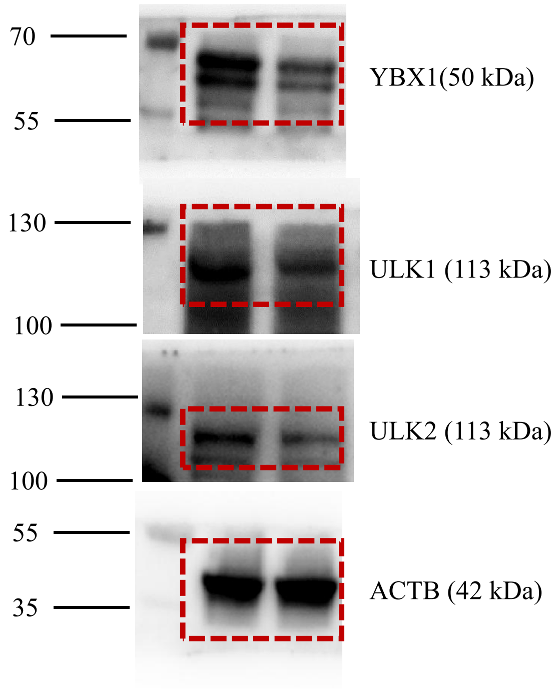

Fig. 3C

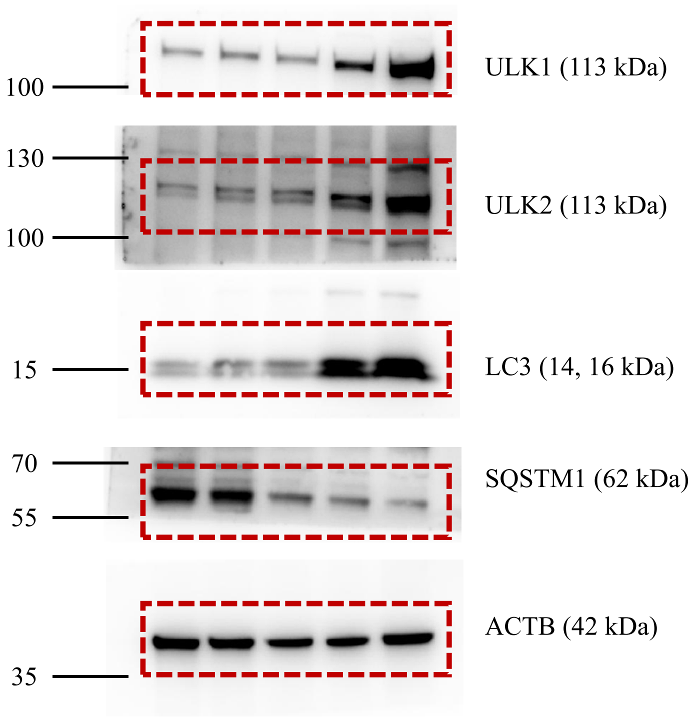

Fig. 3E

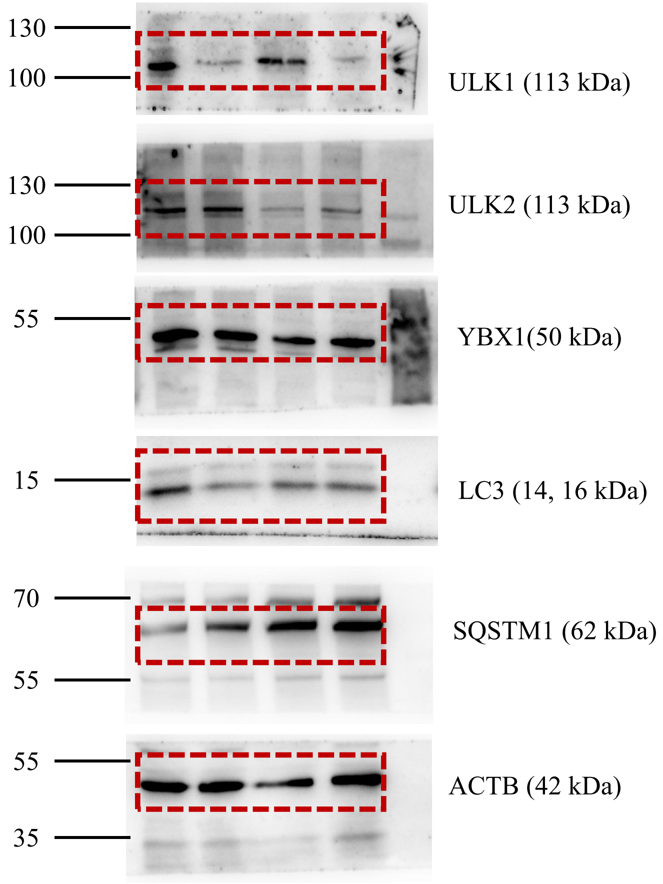

Fig. 4A

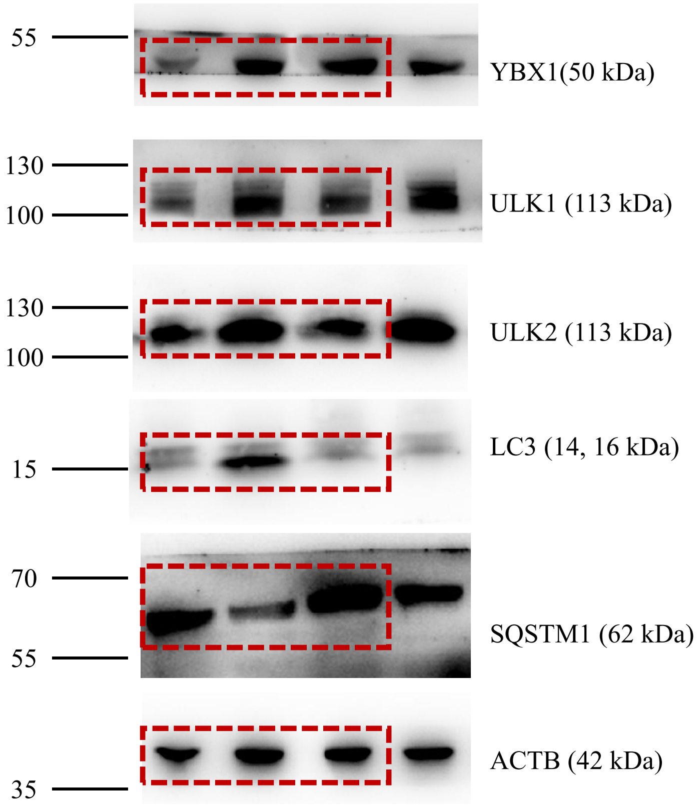

Fig. 5B

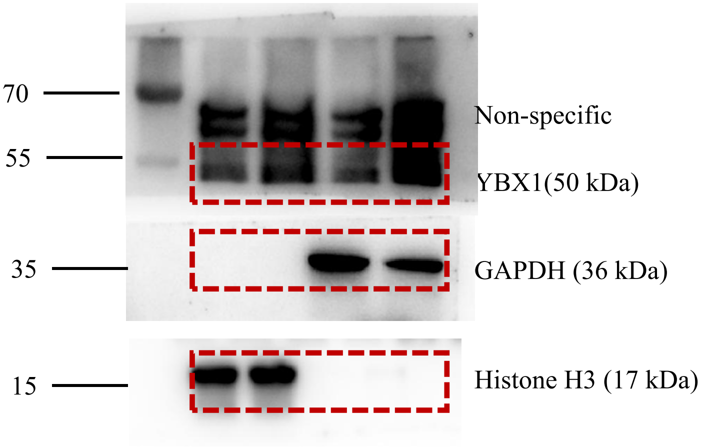

Fig. 7A

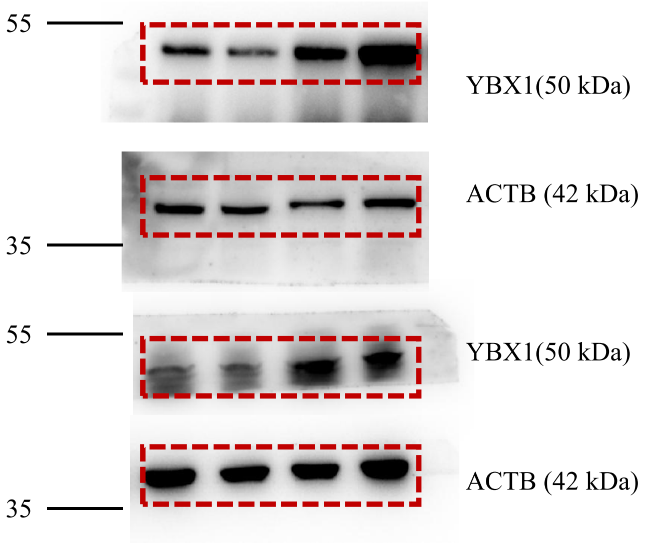

Fig. 5C

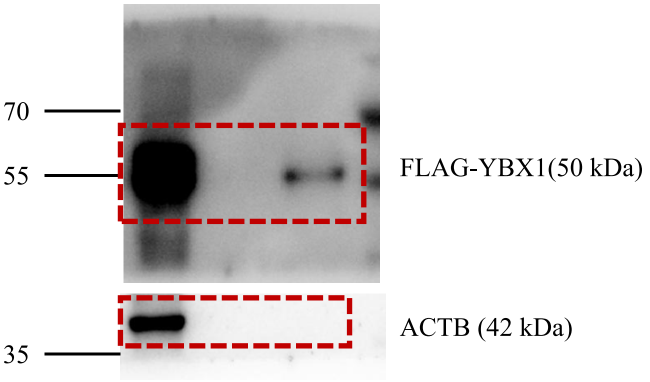

Fig. 7G

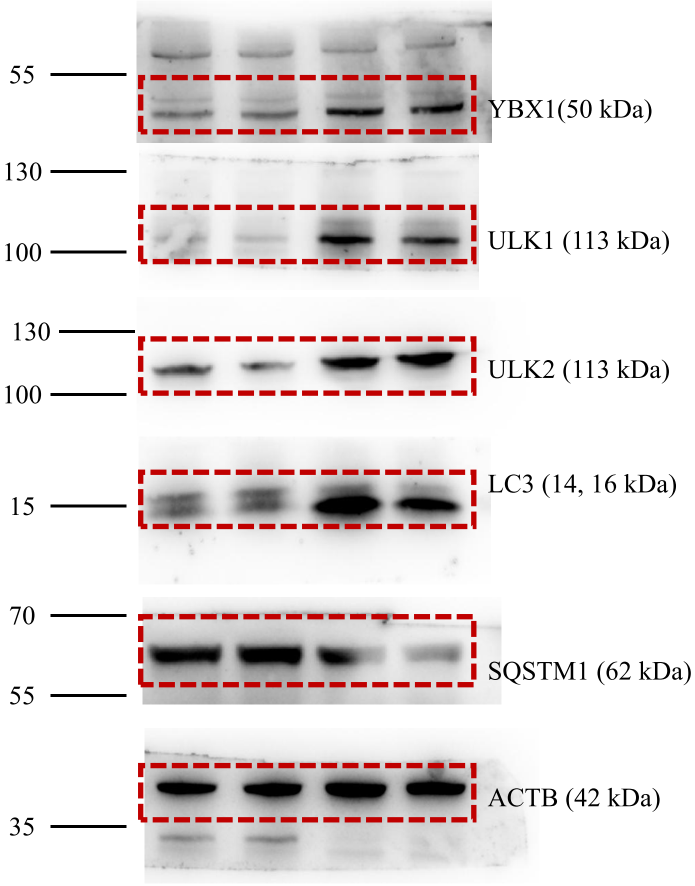

Fig. 7H

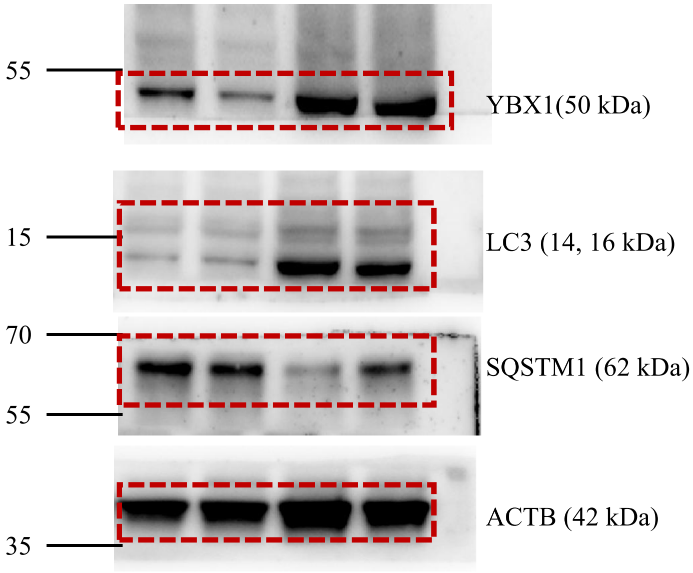

Fig. S1B

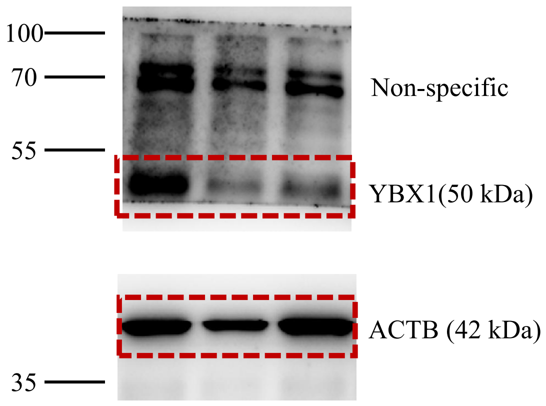

Fig. S1D

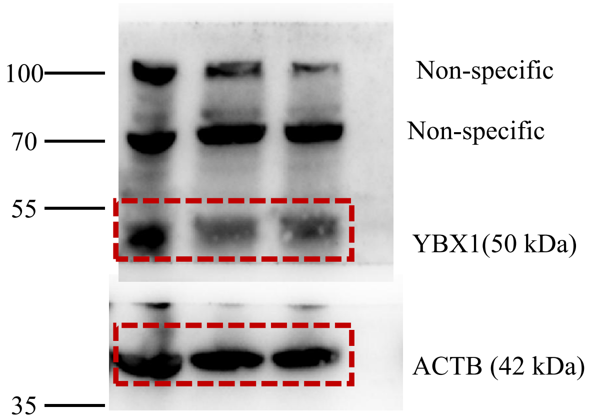

Fig. S2A

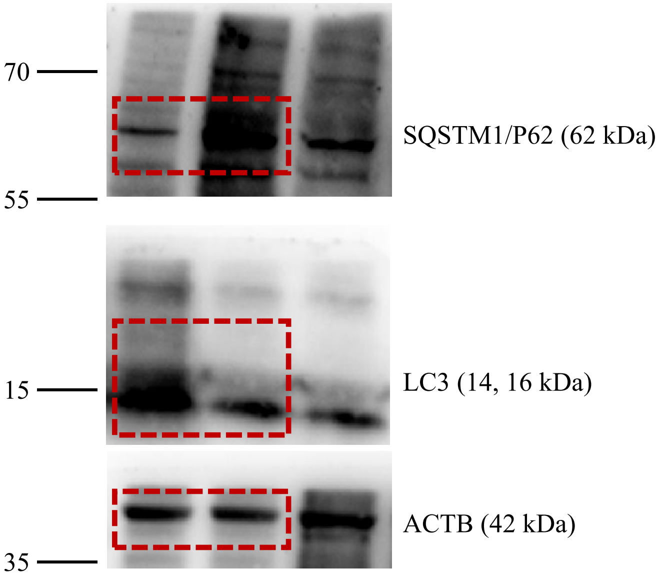

Fig. S3B

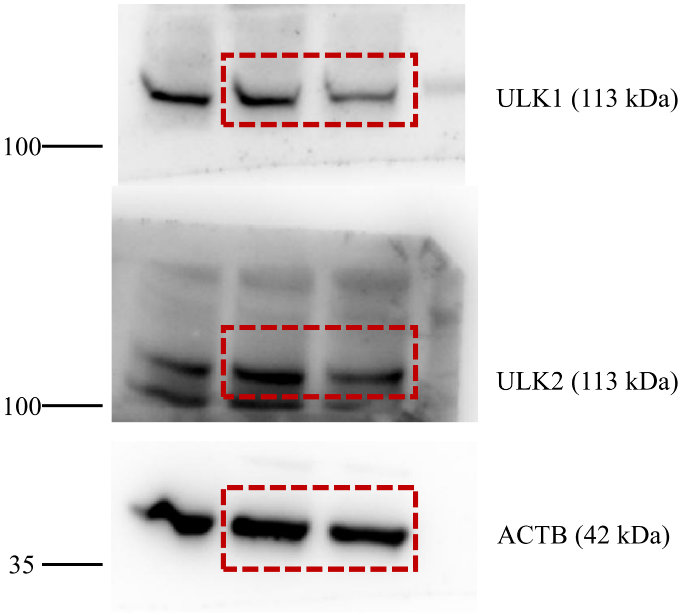

Supplement: Supplementary file 2 — Supplementary Material 2 [file 41419_2023_5564_MOESM2_ESM.pdf]
