# Supplementary figures and images for: Transcriptional and post-transcriptional control of autophagy and adipogenesis by YBX1
Source: Cell Death Dis. 2023 Jan 16;14(1):29. doi: 10.1038/s41419-023-05564-y (PMC9841012; doi:10.1038/s41419-023-05564-y)

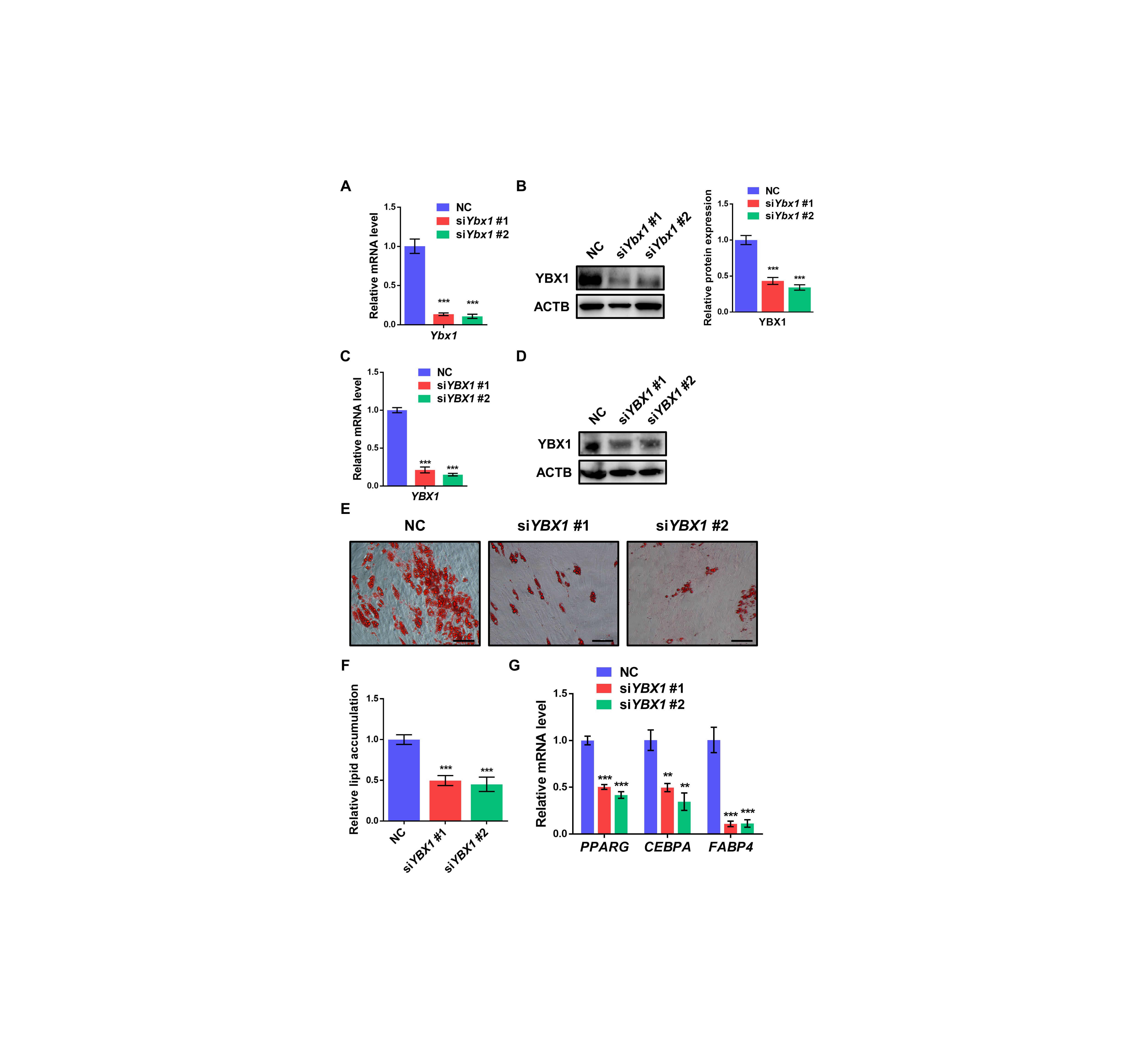

Supplement: Supplementary file 3 — Figure S1 [file 41419_2023_5564_MOESM3_ESM.jpg]

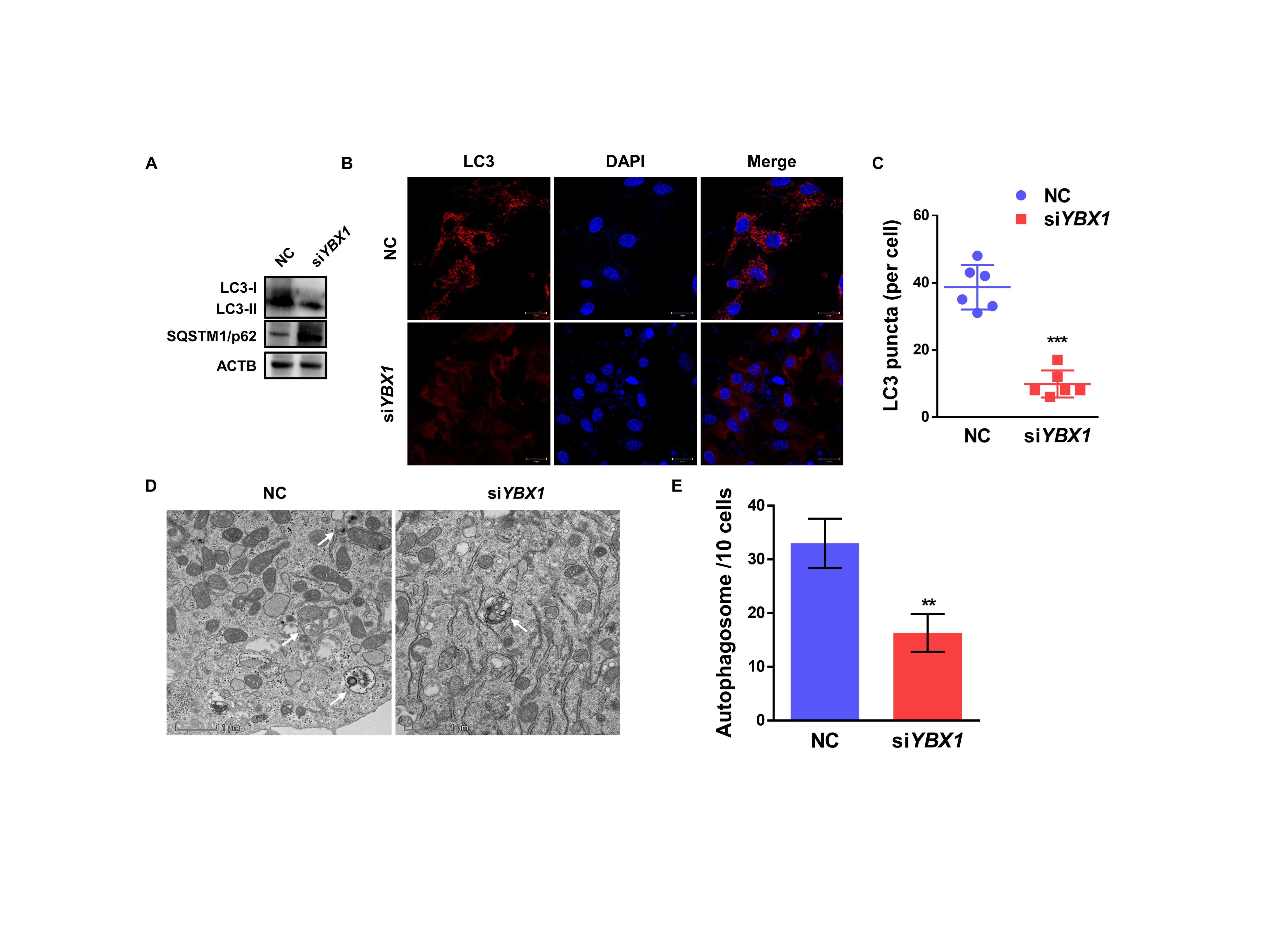

Supplement: Supplementary file 4 — Figure S2 [file 41419_2023_5564_MOESM4_ESM.jpg]

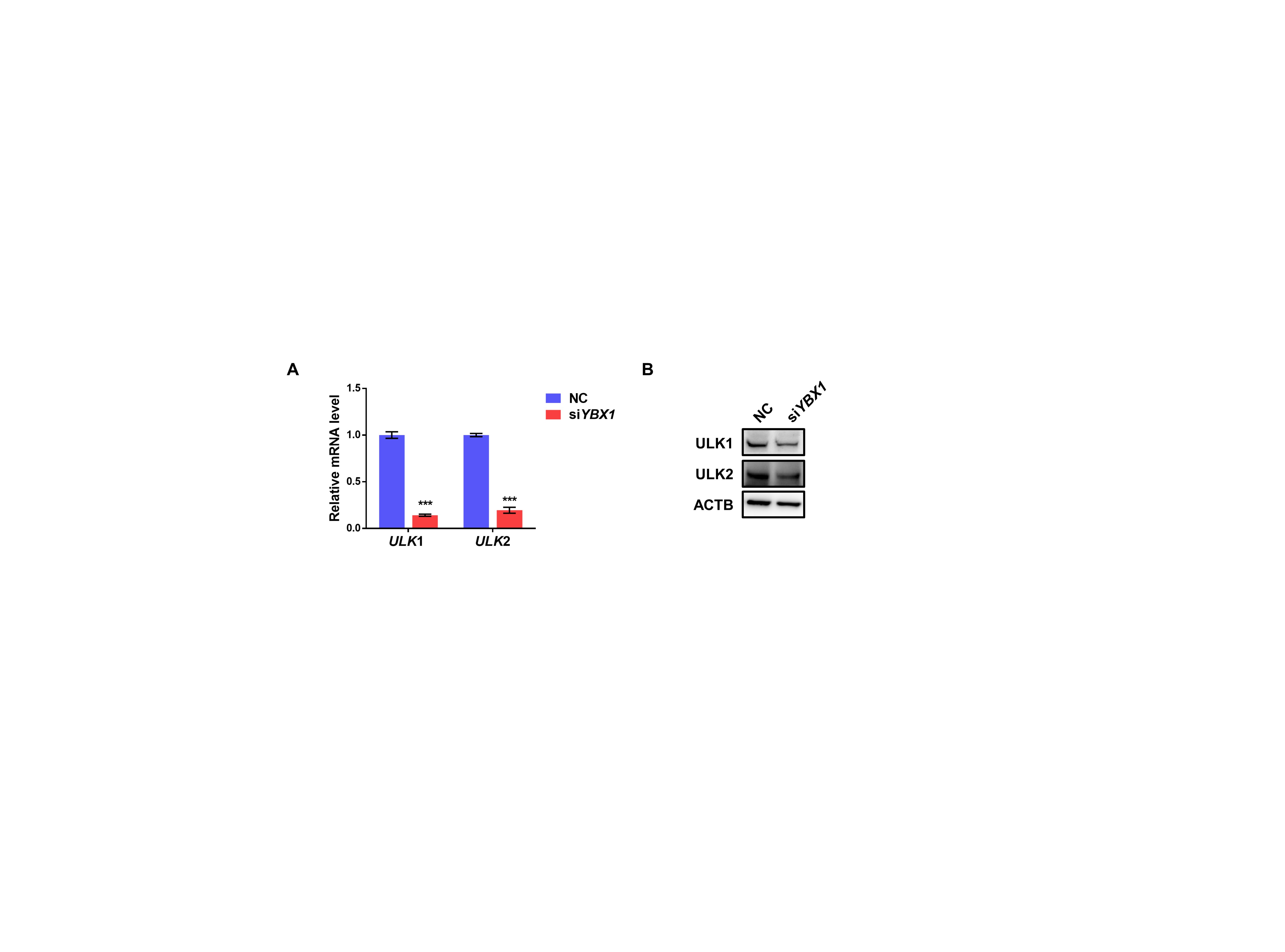

Supplement: Supplementary file 5 — Figure S3 [file 41419_2023_5564_MOESM5_ESM.jpg]
